# An integrative omics-guided druggability analysis of VCX2 in hepatocellular carcinoma using Peruvian natural products

**DOI:** 10.3389/fbinf.2026.1822441

**Published:** 2026-05-18

**Authors:** Luis Daniel Goyzueta-Mamani, Haruna Luz Barazorda-Ccahuana, Mayron Antonio Candia-Puma, Nadia M. Hamdy, Miguel Angel Chávez-Fumagalli

**Affiliations:** 1 Computational Biology and Chemistry Research Group, Vicerrectorado de Investigación, Universidad Católica de Santa María, Arequipa, Peru; 2 Facultad de Ciencias Farmacéuticas, Bioquímicas y Biotecnológicas, Universidad Católica de Santa María, Arequipa, Peru; 3 Biochemistry and Molecular Biology Department Faculty of Pharmacy, Ain Shams University, Cairo, Egypt

**Keywords:** hepatocellular carcinoma (HCC), luteolin-5-O-glucoside, molecular docking, multi-omics integration, natural products, single-cell RNA sequencing, single-cell transcriptomics, VCX2

## Abstract

**Introduction:**

Hepatocellular carcinoma (HCC) is among the deadliest cancers, and current biomarkers offer limited diagnostic and therapeutic utility. Identifying novel druggable targets remains a critical challenge for improving HCC management.

**Methods:**

We implemented an omics-guided computational pipeline integrating single-cell RNA sequencing (scRNA-seq), differential gene expression (DGE) analysis, UMAP clustering, and protein–protein interaction (PPI) network mapping to prioritize candidate genes. Structural characterization of the selected target was performed using AlphaFold-derived models followed by long-timescale molecular dynamics (MD) simulations. Virtual screening of the PeruNPDB (Peruvian Natural Products Database) was conducted using Glide docking, with further evaluation by MM-GBSA and MD-based interaction analyses.

**Results:**

Among prioritized genes (TMBIM4, RGS5, CEACAM7, and VCX2), the cancer/testis antigen VCX2 emerged as a promising candidate due to its aberrant expression and potential involvement in chromosomal instability. MD refinement yielded a stable and ligand-accessible VCX2 conformation. Virtual screening identified luteolin-5-O-glucoside from *Equisetum arvense* as the top ligand (Glide score: −3.949 ± 0.85 kcal/mol; ΔG_bind_: −35.43 ± 1.12 kcal/mol). Despite a modest docking score, consistent with the shallow and polar binding site, MM-GBSA and MD simulations supported a thermodynamically favorable and dynamically stable interaction. Key hydrogen bonds with residues Glu68, Thr63, and Ala61 were maintained within a stabilized polar groove.

**Discussion:**

These findings support VCX2 as a potential molecular target in HCC and highlight luteolin-5-O-glucoside as a promising lead scaffold. This study provides a hypothesis-generating framework that integrates single-cell transcriptomics with structure-based druggability analysis, offering new avenues for targeted therapeutic development in HCC.

## Graphical Abstract

**Figure:**
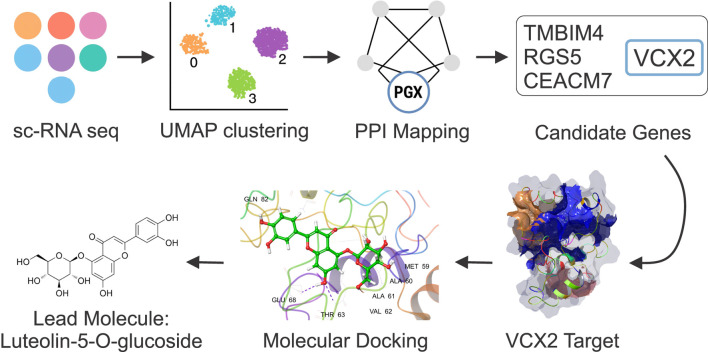

## 1 Introduction

HCC represents approximately 75%–85% of primary liver cancer, causes more than 830,000 fatalities annually (Toh et al., 2023; Siegel et al., 2025), and ranks as the third leading cause of cancer-related mortality worldwide (Guo et al., 2024). Its burden reflects both global incidence and regional disparities: East Asia and Sub-Saharan Africa, where hepatitis B virus (HBV) infection is endemic (Perz et al., 2006; Hammad et al., 2022), show the highest rates (Kafeero et al., 2021), while Western countries report increasing incidence due to metabolic dysfunction-associated steatotic liver disease (MASLD; previously termed non-alcoholic fatty liver disease, NAFLD, and also referred to as metabolic dysfunction-associated fatty liver disease, MAFLD) and obesity (Fan et al., 2017; Eslam et al., 2020; Jiang et al., 2025). Chronic hepatic inflammation, fibrosis, and cirrhosis underlie most cases, driven by viral hepatitis, alcohol, aflatoxin exposure, and metabolic disorders (Fattovich et al., 2004). Clinically, early-stage HCC is often asymptomatic, and current surveillance (imaging plus Alpha-Fetoprotein, AFP) lacks sensitivity and specificity, underscoring the need for more robust biomarkers and targeted strategies (Galle et al., 2019).

At a molecular level, HCC pathogenesis involves a complex interplay of genetic and epigenetic alterations that promote tumor progression, immune evasion, and therapeutic resistance.

Recognized biomarkers, such as AFP (Galle et al., 2019), glypican-3 (GPC3) (Nishida and Kataoka, 2019), and Epithelial cell adhesion molecule (EpCAM), have clinical utility but show limited performance—particularly in early disease—motivating the discovery of more selective targets. Recent single-cell and bulk transcriptomic studies, coupled with network-based prioritization, have highlighted underexplored drivers (e.g., SPP1, AKR1B10), illustrating how integrative omics can reveal mechanistically informative biomarkers (Zheng et al., 2023; Cao et al., 2024; Jin et al., 2024; Tang et al., 2025).

Natural products continue to be a rich source of anti-cancer scaffolds with multi-target activity relevant to HCC biology. Compounds such as curcumin, resveratrol, quercetin, prodigiosin, and berberine modulate apoptosis, angiogenesis, and immune signaling; for example, curcumin attenuates TGF-β–driven EMT/fibrosis (Zhao Y. et al., 2021), resveratrol suppresses VEGF-mediated angiogenesis (Liu et al., 2018), and berberine downregulates GPC3 associated with Wnt/β-catenin signaling (Singh et al., 2023). Hepatoprotective phytochemicals from Silybum marianum (flavonolignans such as silymarin) (Seeff et al., 2015), *Glycyrrhiza glabra* (glycyrrhizin), and *Phyllanthus* species have further motivated biomarker-guided natural-product discovery (Kaur et al., 2017).

Here, we integrate scRNA-seq with pan-cancer transcriptomic profiling to identify Variable Charge X-linked 2 (VCX2) as an underreported, cancer/testis antigen (CTA)–like candidate in HCC. CTAs are typically restricted to immune-privileged germline tissues, and their aberrant expression in tumors provides both diagnostic specificity and therapeutic tractability. We then evaluate VCX2 druggability through structural modeling and molecular dynamics (MD), predict a binding pocket with SiteMap, and perform structure-based screening of the Peruvian Natural Products Database (PeruNPDB) to prioritize ligand candidates. Luteolin-5-O-glucoside emerges as the top binder and is subsequently examined by Glide docking and MM-GBSA. The overall workflow—from scRNA-seq–based prioritization to *in silico* ligand evaluation—is summarized in the Graphical Abstract.

## 2 Materials and methods

### 2.1 Single-cell RNA sequencing data processing and integration

Raw count matrices were imported into R (v4.1.0) (The R Core Team, 2021) and processed using Seurat (v4.0.3) (The R Core Team, 2020). To minimize potential batch effects arising from the use of independently generated scRNA-seq datasets, all datasets were analyzed under a consistent preprocessing pipeline, including quality control filtering, normalization, and dimensionality reduction. For datasets spanning multiple tumor types, raw count matrices were combined using the “MergeSeurat” function to generate a unified Seurat object. Importantly, no aggressive batch integration methods (e.g., canonical correlation analysis or Harmony-based correction) were applied. Instead, analyses were conducted with careful consideration of dataset-specific structure to reduce the risk of artificial clustering driven by technical variation. This strategy prioritizes preservation of biological signal while limiting potential overcorrection artifacts.

For hepatocellular carcinoma (HCC), single-cell RNA-seq data were obtained from the CancerSCEM database, corresponding to the original dataset release (version 1.0). Specifically, we used the dataset associated with NCBI Gene Expression Omnibus (GEO) accession GSE107747 (D’Avola et al., 2018), corresponding to sample HCC-025-01-1A generated using 10X Genomics technology.

The sequential steps of the scRNA-seq analysis pipeline are outlined in the Graphical Abstract and described in detail below to ensure full reproducibility. Single-cell RNA sequencing (scRNA-seq) datasets were collected to characterize transcriptional differences between healthy and cancerous liver tissues. Data from five relatively healthy individuals were obtained from the Gene Expression Omnibus (GEO; GSE115469) (MacParland et al., 2018) (https://www.ncbi.nlm.nih.gov/geo/, accessed 14 February 2024) (Clough et al., 2024), while hepatocellular carcinoma (HCC) samples were retrieved from the CancerSCEM database (https://ngdc.cncb.ac.cn/cancerscem/index, accessed 20 February 2024) (D’Avola et al., 2018; Zeng et al., 2022). To enable broader comparative analyses across tumor microenvironments, additional tumor datasets were included from bladder (Lee H. W. et al., 2020), breast (Gao et al., 2021), colorectal (Lee H.-O. et al., 2020), lung (Lambrechts et al., 2018; Laughney et al., 2020), ovarian (Nelson et al., 2020), pancreatic (Peng et al., 2019), and gastric cancers (Zhang et al., 2020).

The final tumor dataset composition comprised hepatocellular carcinoma (n = 2) (D’Avola et al., 2018), muscle-invasive urothelial bladder cancer (n = 1) (Lee H. W. et al., 2020), triple-negative breast cancer (n = 6) (Gao et al., 2021), breast ductal carcinoma *in situ* (n = 1) (Gao et al., 2021), colorectal cancer (n = 16) (Lee H.-O. et al., 2020), lung adenocarcinoma (n = 21) (Laughney et al., 2020), lung squamous cell carcinoma (n = 7) (Lambrechts et al., 2018), non-small cell lung cancer (n = 7) (Lambrechts et al., 2018), ovarian carcinoma (n = 4) (Nelson et al., 2020), pancreatic ductal adenocarcinoma (n = 24) (Peng et al., 2019), and stomach adenocarcinoma (n = 1) (Zhang et al., 2020).

Gene expression matrices were converted into Seurat objects using the “CreateSeuratObject” function. Cells with fewer than 100 detected genes, more than 25% mitochondrial transcripts, or genes expressed in fewer than three cells were excluded from downstream analysis. Normalization and variance stabilization were performed using the “NormalizeData” function, and batch-associated technical variation, specifically the number of detected unique molecular identifiers (UMIs) and mitochondrial gene expression levels, was regressed using the “ScaleData” function. Highly variable genes were identified with the “FindVariableFeatures” function and subjected to principal component analysis (PCA) using “RunPCA.” The statistical robustness of principal components was evaluated using the “JackStraw” procedure to reduce noise and retain biologically informative components.

Cells were embedded in a nearest-neighbor graph and clustered using the “FindClusters” function, grouping cells with similar transcriptional profiles. Uniform Manifold Approximation and Projection (UMAP) dimensionality reduction was performed using “RunUMAP” to visualize cellular heterogeneity in two-dimensional space. Differential gene expression analyses were conducted using the “FindAllMarkers” and “FindMarkers” functions, applying the Wilcoxon rank-sum test with Bonferroni correction, and genes with adjusted p-values (p_val_adj) < 0.05 were considered statistically significant. Output metrics included adjusted p-values and log-transformed fold change values (avg_logFC). Cell type identities were annotated using the SingleR package (Aran et al., 2019), which computes correlation scores between each query cell and reference transcriptomic atlases, assigning cluster identities based on the highest correlation score.

Functional enrichment analyses were performed using clusterProfiler (Xu et al., 2024) and enrichplot (Yu, 2021), including Gene Ontology (GO) Biological Process, Molecular Function, and Cellular Component categories, as well as Kyoto Encyclopedia of Genes and Genomes (KEGG) pathway enrichment. Visualization outputs—including UMAP dimensional reduction plots, volcano plots, bar plots, dot plots, gene expression heatmaps, and enrichment heatmaps—were generated using the SCpubr package (Blanco-Carmona, 2022), which provides publication-ready wrappers for standard Seurat plotting functions. The full R script used for scRNA-seq preprocessing, dataset integration, clustering, annotation, enrichment analysis, and SCpubr-based visualization is publicly available through the project repository referenced in the Data Availability Statement, while detailed usage of SCpubr functions follows the official package documentation (https://enblacar.github.io/SCpubr-book/, accessed 25 January 2025).

### 2.2 Protein-protein interaction (PPI) network and tissue-specific expression analysis

To better understand the biological context of candidate biomarkers, protein–protein interaction (PPI) networks were constructed using the STRING database (v12.0) (https://string-db.org, accessed 20 April 2024) (Szklarczyk et al., 2015). Candidate biomarkers identified from differential expression analyses were first mapped to their corresponding protein products prior to PPI construction to ensure accurate network representation. Only interactions with high confidence scores (≥0.70) were retained to minimize false-positive associations and increase biological reliability. Network visualization was performed in Cytoscape (v3.9.1) (Shannon et al., 2003) following default parameters; however, node sizes and color gradients were manually adjusted to reflect Maximal Clique Centrality (MCC)-derived scores and inferred biological relevance. Topological analysis and centrality metrics were computed using the cytoHubba plugin (Chin et al., 2014), applying the MCC algorithm to identify highly interconnected hub proteins potentially relevant to hepatocarcinogenesis.

Functional enrichment of the interaction network was conducted using the StringApp plugin (Gorodkin et al., 2019; Szklarczyk et al., 2015), which retrieves Gene Ontology (GO) annotations across Biological Process, Molecular Function, and Cellular Component categories, as well as Kyoto Encyclopedia of Genes and Genomes (KEGG) pathway information directly from STRING. In addition to pathway enrichment, co-expression evidence integrated within STRING was considered to support functional associations among candidate proteins. To assess tissue-specific expression patterns and strengthen biological validation, candidate genes were cross-validated using the TISSUES database (v2.0) (https://tissues.jensenlab.org/Search, accessed 25 April 2024), which integrates transcriptomic and proteomic evidence to provide confidence scores for tissue-specific gene expression (Palasca et al., 2018).

### 2.3 Structural refinement of VCX2: molecular dynamics and protein preparation

Prior to molecular dynamics (MD) refinement, multiple structure-prediction approaches were employed to assess the conformational tendencies and define the ordered core region of VCX2 (139 amino acids; UniProt ID: Q9H322). The amino acid sequence was retrieved in FASTA format from the UniProt database (accessed 5 March 2024), and an initial three-dimensional model was obtained from the AlphaFold Protein Structure Database v4, which provides AlphaFold2-based structural predictions of the human proteome (average pLDDT = 62.6). Secondary structure and disorder propensities were evaluated using PSIPRED v4.0 and DISOPRED3, which provided residue-level probabilities for α-helical, β-strand, and coil conformations. Threading and meta-threading analyses were conducted with Phyre2 and I-TASSER using default parameters to identify structurally compatible templates. Independent AI-based structural predictions were also generated using Chai-1 and Boltz-2 under standard inference settings to estimate pTM and pLDDT confidence scores. Coordinates from all predictors were compared to identify consensus secondary-structure elements and to define the structured core region retained for subsequent MD simulations.

Protonation states were initially assigned at pH 7.4 using PDB2PQR and PropKa prior to simulation setup. Molecular dynamics simulations were performed using GROMACS 2022.4 with the CHARMM36m force field. The protein was solvated in a cubic TIP3P explicit water box and neutralized with 0.15 M Na^+^/Cl^−^ ions to approximate physiological ionic strength. Solvent equilibration was confirmed during the NPT stage by monitoring system density with gmx energy, which stabilized at approximately 0.98 g/mL at 309.65 K prior to annealing and production phases (Supplementary Figure S6). Structural refinement followed a multi-stage protocol consisting of steepest-descent energy minimization (5,000,000 steps) to eliminate steric clashes, followed by simulated annealing comprising 12 cycles of 500 ps each, in which the system was gradually heated to 500 K and cooled back to 300 K, yielding a total annealing time of 50 ns. Equilibration was subsequently conducted under NVT conditions at 300 K for 5 ns with positional restraints applied to heavy atoms to allow solvent adaptation. A 1000-ns (1 μs) unrestrained production simulation was then performed under NPT conditions at 309 K (approximately 36 °C) and 1 bar using the V-rescale thermostat and Parrinello–Rahman barostat. Three independent replicates with randomized initial velocities were carried out to capture conformational variability and ensure reproducibility.

Structural stability and convergence were assessed through root mean square deviation (RMSD), root mean square fluctuation (RMSF), and total energy profiles across all replicates. Principal component analysis (PCA) was performed on concatenated trajectories, and eigenvalue decay plots (Supplementary Figure S2A) demonstrated consistent variance distribution among principal components. The full trajectory PCA (Supplementary Figure S2B) revealed distinct metastable conformational states that were dynamically stable, well-separated, and long-lived throughout the simulation, supporting global conformational convergence. The refined models were validated using ProSa-web (https://prosa.services.came.sbg.ac.at/prosa.php) and PDBsum (https://www.ebi.ac.uk/thornton-srv/databases/pdbsum/), accessed 8 March 2025. ProSa analysis yielded a global Z-score within the range of experimentally determined structures of comparable size, while Ramachandran plot assessment from PDBsum indicated that more than 95% of residues were located in favored regions, 4.8% in additionally allowed regions, and 0% in disallowed regions, representing an improvement over the original AlphaFold prediction (2.8% disallowed residues). Knowledge-based energy plots and per-residue energy profiles further confirmed stereochemical integrity.

The MD-refined structure was subsequently processed in Schrödinger Maestro v12.8.117 (Release, 2020) using the Protein Preparation Wizard. Bond orders were corrected based on Chemical Components Dictionary data, hydrogen atoms were added, and side chains and loop regions were rebuilt using Prime. Protonation states were predicted at physiological pH (7.0 ± 0.2) using Epik, hydrogen-bonding networks were optimized, and all solvent molecules and hetero-ligands located more than 3.0 Å from heteroatoms were removed. A final restrained energy minimization was performed using the OPLS4 force field (Lu et al., 2021), generating a high-quality receptor model suitable for downstream binding site prediction (SiteMap) and molecular docking simulations. Electrostatic potential surfaces of the refined structure were also computed using the Protein Electrostatics workflow in Maestro with OPLS4 charges and a Poisson–Boltzmann implicit solvent model to characterize charge distribution at physiological pH.

### 2.4 Virtual screening with the Peruvian natural products database (PeruNPDB)

A structure-based virtual screening was performed using phytochemicals from the PeruNPDB (https://perunpdb.com.pe, accessed 15 April 2024) (Barazorda-Ccahuana et al., 2023), a curated collection of small molecules derived from native Peruvian biodiversity and encoded in SMILES format (Weininger, 1988). A total of 280 SMILES-encoded compounds were retrieved from the official PeruNPDB web server (https://perunpdb.com.pe, accessed 15 April 2024) (Barazorda-Ccahuana et al., 2023) and all compounds were included in the virtual screening workflow. Ligand preprocessing was conducted using OpenBabel (v3.1.1), integrated within the Python Prescription Virtual Screening Tool (Dallakyan and Olson, 2015), where SMILES strings were converted into three-dimensional conformations followed by geometry optimization and energy minimization to generate structurally consistent ligand models suitable for docking.

The molecular dynamics-refined VCX2 structure was used as the receptor for docking simulations. Virtual screening was performed in PyRx (v0.9.8) employing AutoDock Vina (v1.2.5) (Trott and Olson, 2010). Docking grid parameters were defined using the “Run AutoGrid” option, and simulations were executed under the Run AutoDock module using the Lamarckian Genetic Algorithm with an exhaustiveness value of 20 to enhance sampling robustness. For reproducibility and comparability across compounds, only the lowest-energy binding pose for each ligand was retained for subsequent analysis.

To standardize binding affinity results across the compound library, docking scores were normalized using Z-score transformation. A statistical threshold of ±1.645, corresponding to p < 0.05 under a normal distribution assumption, was applied to identify significantly favorable interactions relative to the dataset mean. The distribution of binding affinities and standardized scores was visualized using violin plots generated in GraphPad Prism (v10.0.2; GraphPad Software, San Diego, CA, United States; http://www.graphpad.com), enabling comparative evaluation of ligand performance across the screened phytochemical set.

### 2.5 Binding site prediction, grid generation, and ligand preparation

Following structural refinement of the VCX2 model, potential ligand-binding pockets were identified using the SiteMap module implemented in Schrödinger Maestro (v12.8.117). The algorithm predicted and ranked the top five binding sites on the protein surface according to composite site score, pocket volume, enclosure, solvent exposure, and the balance between hydrophobic and hydrophilic character. To improve pharmacophoric focus and eliminate peripheral noise, site maps were cropped at a 4.0 Å radius from the nearest site point. The highest-scoring binding pocket, which was consistent with conformations stabilized during molecular dynamics simulations and supported by principal component analysis, was selected for subsequent docking studies.

A receptor grid was generated using the Receptor Grid Generation wizard in Glide (Schrödinger Suite), centered on the centroid of the selected SiteMap pocket. The grid box dimensions were adjusted to fully enclose the predicted binding cavity while minimizing excess volume to avoid nonspecific docking poses. No positional constraints or pharmacophore constraints were applied during grid preparation, allowing unbiased ligand accommodation within the defined pocket.

For reproducibility, the top-ranked ligands identified during virtual screening were prepared in Schrödinger Maestro (v12.8.117) using the LigPrep wizard. Protonation states and tautomeric forms were generated with Epik at pH 7.0 ± 2.0 to approximate physiological conditions, and stereoisomer enumeration was capped at a maximum of 32 stereoisomers per compound to ensure broad conformational coverage while maintaining computational tractability. All ligand structures were energy-minimized using the OPLS4 force field prior to docking to ensure geometrically optimized input conformations suitable for high-accuracy binding pose prediction.

### 2.6 Molecular docking, molecular dynamics and MM-GBSA binding free energy calculations

Structure-based molecular docking was performed using the Glide module (Schrödinger Suite v12.8.117) within the receptor grid defined by the top-ranked SiteMap binding pocket. A multi-tiered precision protocol was implemented to improve pose discrimination and scoring robustness. Initially, Standard Precision (SP) docking was applied to filter sterically unfavorable conformations and identify plausible ligand orientations within the binding cavity. Surviving candidates were subsequently redocked using Extra Precision (XP), which incorporates enhanced sampling and a refined scoring function to more rigorously evaluate binding orientations and interaction energetics. During docking, enhanced sampling was enabled, and a van der Waals scaling factor of 0.80 was applied to nonpolar ligand atoms to improve discrimination of sterically constrained poses while maintaining conformational flexibility. Docking results were ranked according to GlideScore, and top-ranked complexes were analyzed in Maestro using the Interaction Diagram tool to characterize hydrogen bonds, salt bridges, hydrophobic contacts, and π–π stacking interactions within the VCX2 binding pocket.

In the absence of experimentally validated ligands for VCX2, no redocking-based calibration or benchmarking against a reference set was performed. Instead, docking results were interpreted comparatively within the screened compound library. To address the known limitations of empirical scoring functions, particularly in solvent-exposed and polar binding environments, docking outcomes were further refined through molecular dynamics simulations and Molecular Mechanics/Generalized Born Surface Area (MM-GBSA) binding free energy calculations (Equations 1-3).

To complement docking and evaluate binding energetics under thermodynamically realistic conditions, MM-GBSA calculations were performed using the Prime module (v4.8, Schrödinger Suite) with the VSGB 2.0 implicit solvation model and the OPLS4 force field. Structural snapshots of the protein–ligand complexes were extracted from the equilibrated portion of the 200 ns molecular dynamics trajectories, specifically from the final 10 ns, based on RMSD stabilization and total energy convergence to ensure equilibrium sampling.

Binding free energy (ΔG_bind_) was calculated using the single-trajectory MM-GBSA approach, according to the following equations:

$$\Delta G_{\text{bind}}=G_{\text{complex}}-\left(G_{\text{protein}}+G_{\text{ligand}}\right)$$ (1)

Where each free energy term G is defined as:

$$G=E_{\text{MM}}+G_{\text{solv}}+G_{\text{SA}}$$ (2)

Thus,

$$\Delta G_{\text{bind}}=\Delta E_{\text{MM}}+\Delta G_{\text{solv}}+\Delta G_{\text{SA}}$$ (3)

ΔE_MM_: Difference in molecular mechanics energies (bonded, van der Waals, electrostatics) between the bound and unbound states.
ΔG_solv_: Solvation free energy difference calculated using the VSGB 2.0 implicit solvation model.
ΔG_SA_: Nonpolar solvation contribution estimated from solvent-accessible surface area (SASA).

Ligand strain energy and interaction energy components were also computed to account for conformational penalties and steric effects upon binding.

Per-residue free energy decomposition was carried out using the Prime Energy Visualizer to identify hotspot residues contributing most significantly to ligand stabilization within the VCX2 pocket. For the VCX2–Luteolin-5-O-glucoside complex, additional molecular dynamics simulations were conducted in Schrödinger Maestro v12.8 using the OPLS4 force field. The system was solvated in a TIP3P explicit water box, neutralized with 0.15 M NaCl, and simulated under NPT conditions at 309.65 K and 1 atm for 200 ns across three independent replicates to ensure reproducibility. Root mean square deviation (RMSD) and root mean square fluctuation (RMSF) analyses were performed to assess complex stability and residue-level flexibility.

## 3 Results and discussions

### 3.1 Identification and prioritization of candidate molecular targets in hepatocellular carcinoma

To identify liver cancer–specific biomarkers with potential therapeutic relevance, we integrated single-cell RNA sequencing (scRNA-seq) data with differential gene expression (DGE) and protein–protein interaction (PPI) network analyses. This multi-omics approach aimed to capture both cell-type-specific transcriptional changes and molecular interactions driving hepatocellular carcinoma (HCC) progression. DEGs were filtered using Bonferroni-adjusted *p* < 0.05 and |log_2_FC| > 1, and subsequently ranked by biological relevance through centrality metrics within the PPI network. Candidate hub genes were validated across tumor datasets to assess specificity and oncogenic consistency.

Among the prioritized genes—TMBIM4, RGS5, CEACAM7, and VCX2—the Variable Charge X-linked 2 (VCX2) gene emerged as particularly intriguing. Its restricted expression in germline tissues and reactivation in HCC suggested a cancer/testis antigen–like profile. Given this dual specificity and its potential link to chromosomal instability, VCX2 was selected for in-depth modeling and druggability analysis, forming the rationale for the subsequent computational and structural investigations.

To provide a clearer rationale for target prioritization, candidate genes were evaluated through an integrative framework combining transcriptomic significance (adjusted p-value and log_2_ fold-change), network topology (MCC-based centrality within the PPI network), cell-type specificity derived from single-cell RNA sequencing, and biological context. Within this framework, VCX2 was not selected based on absolute expression levels alone, but rather on its distinct cancer/testis antigen-like profile, disease-associated expression pattern, and network connectivity. This combination of features suggests a more favorable balance between biological relevance and therapeutic selectivity compared to more broadly expressed candidates, supporting its prioritization for downstream structural and druggability analyses.

**FIGURE 1:**
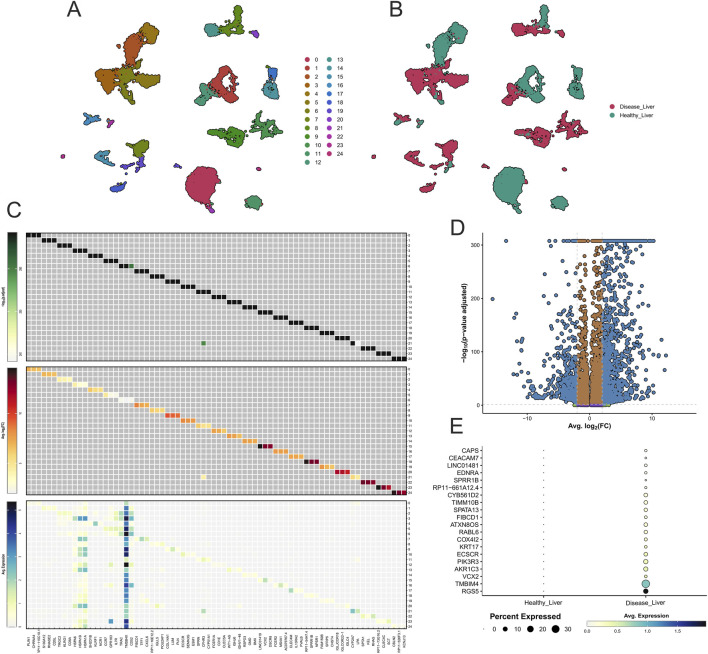
Integrated single-cell analysis of liver cell populations and transcriptional changes in health and disease. **(A)** UMAP projection of transcriptionally defined clusters from single-cell RNA-seq data. Numbers denote unsupervised clusters (0–24). **(B)** UMAP overlay of healthy (green) versus diseased (red) samples highlights transcriptional divergence and shifts in cellular composition. **(C)** Heatmaps depict gene relationships and cluster-specific markers, with the upper heatmap showing correlations among the top three genes per cell cluster. **(D)** Volcano plot of differential expression between healthy liver and HCC. **(E)** Dot plot summarizes expression dynamics across liver cell types, with color intensity representing average expression and dot size reflecting the percentage of expressing cells. For corresponding Gene Ontology and pathway enrichment analyses of these differentially expressed genes, see Figure 2 (panels B–E).

**FIGURE 2:**
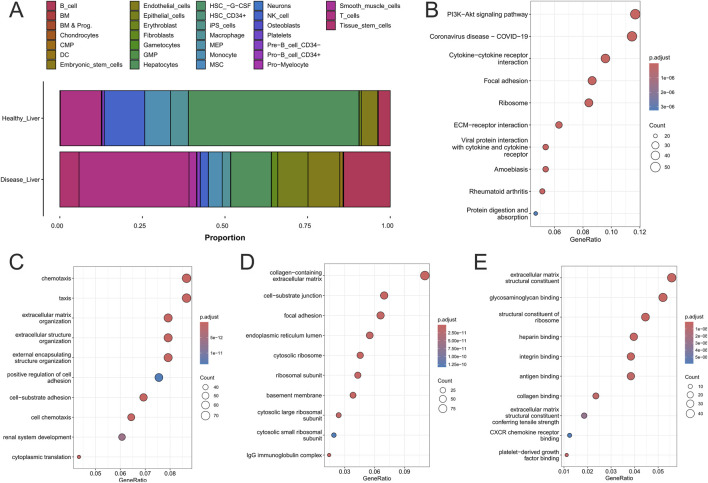
Comprehensive analysis of molecular, cellular, and functional alterations in healthy and diseased liver tissues. **(A)** Proportional representation of cell types in healthy and diseased liver tissues. **(B)** KEGG pathway enrichment analysis. **(C)** Biological process enrichment. **(D)** Cellular component enrichment. **(E)** Molecular function enrichment.

**FIGURE 3:**
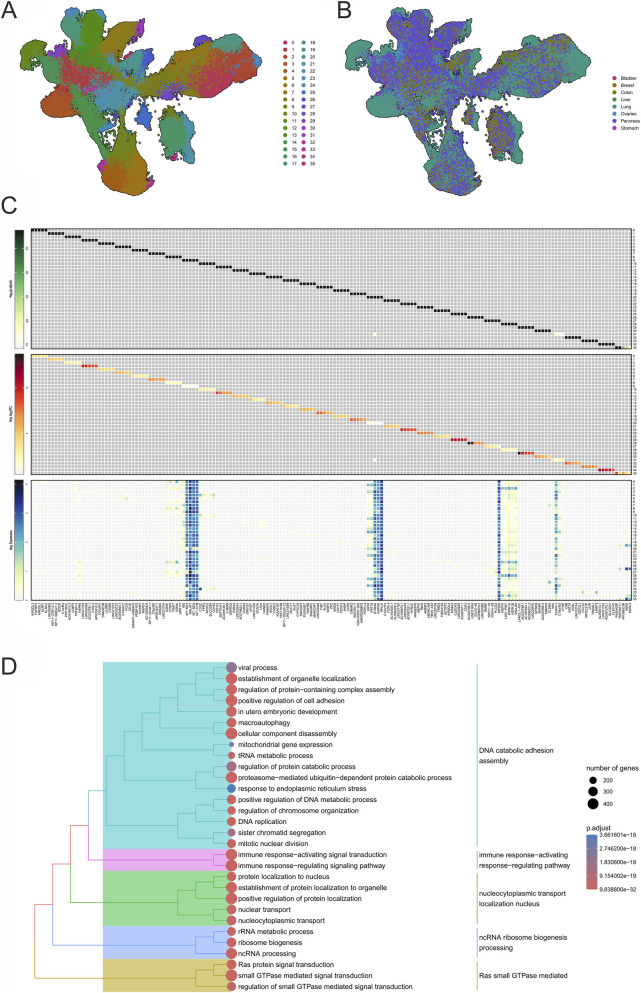
Comprehensive analysis of cancer biomarker expression and pathway enrichment across multiple cancer types. **(A, B)** present clustering maps derived from eight cancer datasets, identifying transcriptional patterns and distinct gene expression clusters associated with specific cancer types. **(C)** Showcases a heatmap highlighting the top markers per cluster, illustrating their expression levels and functional roles across cancer datasets. **(D)** Displays a tree plot of enrichment analysis for all biomarkers in the dataset, with pathways color-coded to represent their involvement in biological processes.

**FIGURE 4:**
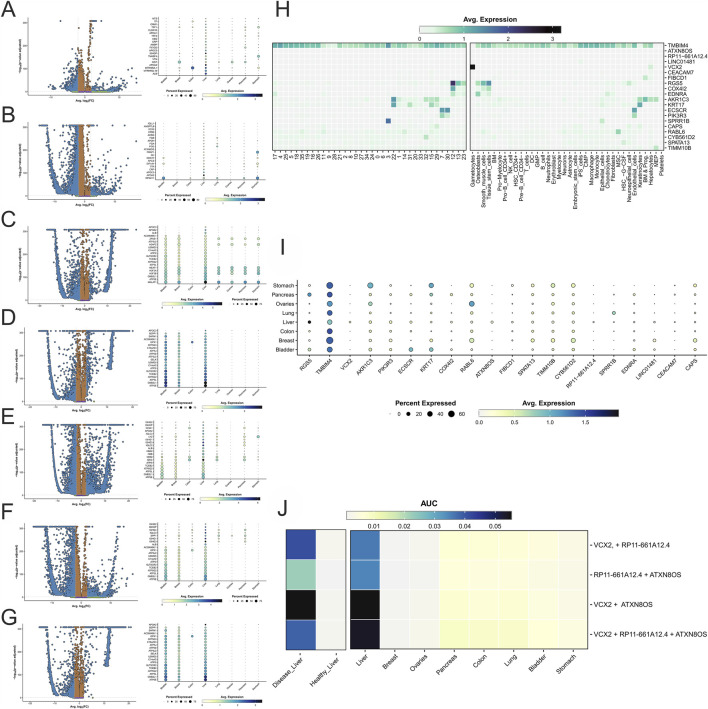
The volcano plots show differentially expressed genes in liver cancer than **(A)** bladder, **(B)** breast, **(C)** colon, **(D)** lung, **(E)** ovaries, **(F)** pancreas, and **(G)** stomach cancers. The dot plots represent the results of the top 20 genes per comparison. **(H)** Heatmap showing the results for each cluster, cell annotations for the genes expressed differently in HCC vs. healthy liver, and **(I)** the dot plot of the same genes by cancer organ dataset. **(J)** Enrichment heatmap for a combination of markers against healthy and diseased liver datasets and the eight cancer tissue datasets.

**FIGURE 5:**
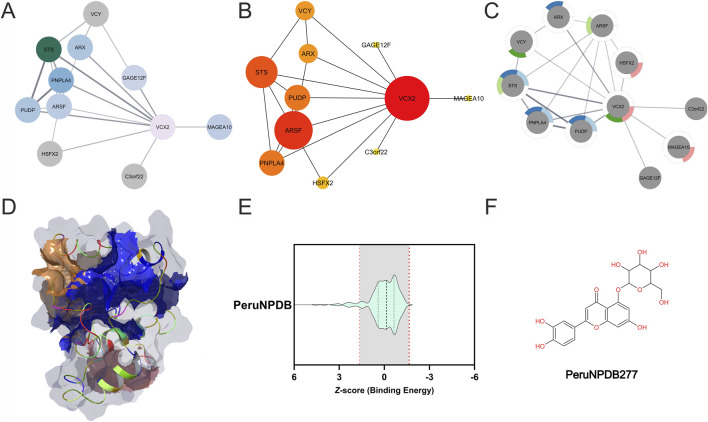
Network analysis and therapeutic exploration of VCX2. (Top) Three network diagrams illustrating VCX2’s molecular interactions. **(A)** Node-link diagram depicting VCX2 (pink) and its associated genes. **(B)** Centralized network emphasizing key partners. **(C)** Expanded interaction network, where color gradients highlight shared genes or proteins. **(D)** Identification of druggable pockets in VCX2. **(E)** Molecular docking results and binding affinity analysis (Z-score) to assess candidate compounds. **(F)** Structural representation of PeruNPDB277 (Luteolin-5-O-glucoside).

**FIGURE 6:**
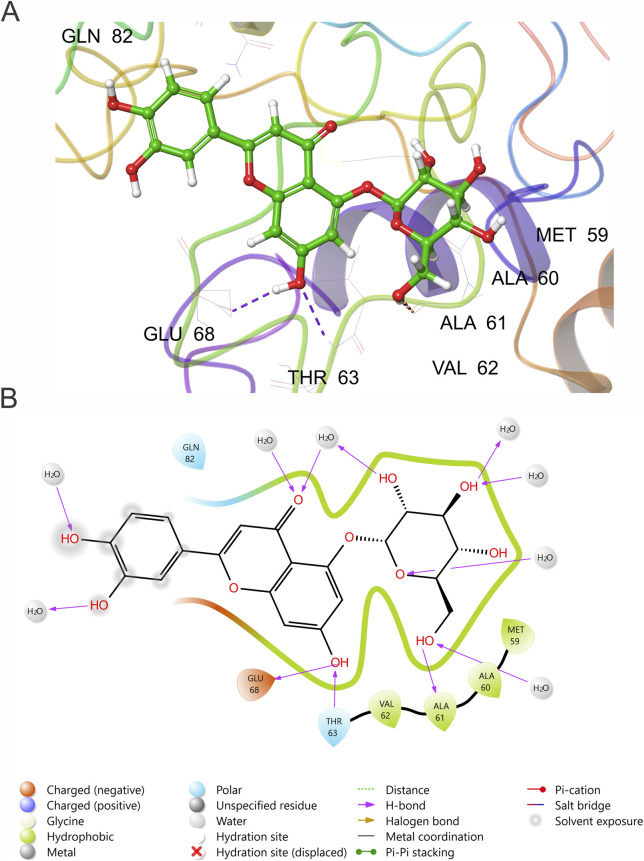
Molecular interactions of Luteolin-5-O-glucoside with VCX2. **(A)** MD-refined 3D binding pose showing persistent hydrogen bonds (purple dashed lines) with Glu68, Thr63, and nearby structured waters within the polar VCX2 pocket. **(B)** 2D interaction map highlighting the main electrostatic and hydrogen-bond contacts contributed by Glu68, Thr63, Ala60, Ala61, Val62, Met59, and Gln82, consistent with the shallow and hydrophilic nature of the site.

Our integrative transcriptomic analysis highlights VCX2 as a previously underexplored candidate with cancer/testis-like features in HCC. Differential expression patterns observed in scRNA-seq datasets suggest a potential disease-associated signal, while pan-cancer comparisons indicate a context-dependent expression profile enriched in liver malignancy. Although further validation in independent and clinically matched cohorts is required to confirm its biomarker relevance, structural modeling of VCX2 revealed a potentially druggable binding pocket that enabled structure-based screening of the PeruNPDB natural products library. From this screen, Luteolin-5-O-glucoside emerged as the top-ranked candidate (Docking score: –3.949 ± 0.85 kcal/mol; Δ_Gbind_: –35.43 ± 1.12 kcal/mol). Together, these findings provide a hypothesis-generating framework that integrates single-cell transcriptomics and structural druggability analysis to prioritize emerging molecular targets for therapeutic exploration in HCC (Figures 1–6). Within this context, VCX2 may function as a conformationally dynamic regulatory protein in tumor-associated states, with ligand binding potentially modulating transient interaction surfaces rather than a classical catalytic function.

Although multiple candidate genes were identified through differential expression and network analysis, VCX2 was selected for structural and druggability assessment based on a combination of biological specificity and target tractability. Unlike other candidates such as TMBIM4 and RGS5, which are more broadly expressed and functionally conserved across tissues, VCX2 belongs to the class of cancer/testis antigens, characterized by restricted expression in normal tissues and aberrant activation in cancer.

This expression pattern suggests a potentially favorable therapeutic window and reduced risk of off-target effects. Furthermore, structural analysis revealed that VCX2 contains a solvent-accessible, dynamically persistent binding groove capable of supporting small-molecule interactions, supporting its prioritization for structure-based investigation. Together, these features position VCX2 as a biologically relevant and structurally actionable target, despite not being the most highly expressed gene among the candidates.

### 3.2 Transcriptional reprogramming and cellular heterogeneity in hepatocellular carcinoma

HCC progression involves a complex interplay of fibrosis, angiogenesis, immune dysregulation, and impaired apoptosis. To investigate these mechanisms, we applied scRNA-seq to generate a high-resolution transcriptional landscape of hepatic cell populations. UMAP projections (Figure 1A) revealed multiple transcriptionally distinct clusters, with each numbered group representing a population of transcriptionally similar cells identified through unsupervised clustering. Although specific cell-type annotations were not yet assigned at this stage, the distinct clustering patterns laid the groundwork for subsequent identity classification and biomarker prioritization. Overlay analysis of healthy (green) and diseased (red) samples (Figure 1B) showed marked compositional shifts, with HCC tissues exhibiting expanded populations in specific clusters. These changes are consistent with the fibrotic remodeling and immune activation characteristic of hepatic tumorigenesis and are further explored through pathway and functional enrichment analyses in the following section.

HCC progression involves chronic inflammation, immune dysregulation, fibrogenesis, and extracellular matrix (ECM) remodeling, which together establish a tumor-permissive microenvironment (Carloni et al., 2014; Ali et al., 2021). To capture these transitions, we compared the cellular and transcriptional architecture of healthy and diseased liver tissues (Figure 2).

Figure 1C shows a heatmap summarizing the top three marker genes per cluster. The upper panel highlights co-expression patterns, revealing a strong correlation between TMBIM4 and RGS5, genes implicated in apoptosis inhibition and vascular remodeling (Rojas-Rivera and Hetz, 2015). The middle panel reports statistical significance (adjusted p < 0.05), with markers such as TMBIM4, RGS5, and CEACAM7 displaying robust shifts. CEACAM7, normally tumor-suppressive, exhibited aberrant expression in HCC (Arnold et al., 2014), consistent with its role in malignant transformation (Beauchemin and Arabzadeh, 2013). The lower panel illustrates log_2_ fold-changes, where TMBIM4, RGS5, and Cytochrome B5 Reductase 3 (CYB5R3) were upregulated, indicating their roles in apoptosis resistance, oxidative stress adaptation, and metabolic reprogramming (Rodríguez López, 2021; Im et al., 2024). Downregulated hepatocyte-specific genes reflected impaired metabolic capacity, while VCX2 showed unexpectedly low expression, underscoring the need for broader contextual analyses (Jakobsen et al., 2021).

The volcano plot in Figure 1D provides a global overview of differential expression, with TMBIM4 and RGS5 among the most significantly upregulated markers, supporting their roles in survival, immune evasion, and vascular remodeling. Conversely, downregulated genes clustered on the left side of the distribution were enriched in hepatocyte metabolic pathways that are suppressed during tumorigenesis (Gilgenkrantz et al., 2025).

Finally, Figure 1E illustrates overall gene expression patterns across healthy and diseased liver samples. Dot size reflects the percentage of cells expressing each gene, while color intensity indicates mean expression. Among the genes analyzed, TMBIM4, RGS5, and VCX2 displayed markedly higher average expression in the diseased condition, consistent with their enrichment in fibroblasts, macrophages, T cells, and B cells observed in the cluster-level analysis. Notably, TMBIM4 showed marked enrichment in tumor-associated fibroblasts and immune cells, suggesting a role in apoptosis resistance within the fibrotic and inflammatory microenvironment characteristic of HCC (Chen et al., 2023). The functional relevance of these transcriptional changes was further supported by Gene Ontology and KEGG enrichment analyses shown in the next figure.

### 3.3 Pathway enrichment and functional relevance of differentially expressed genes

As shown in Figure 2A, bar plots show the relative abundance of major hepatic cell types. Healthy livers were primarily composed of hepatocytes (green), with smaller contributions from macrophages (blue), endothelial cells (yellow), fibroblasts (brown), T cells (dark pink), and B cells (light pink). Diseased tissues displayed a sharp decline in hepatocytes alongside expansion of fibroblasts, macrophages, and immune cells, reflecting fibrotic remodeling and chronic inflammation typical of HCC (Yang et al., 2003; Capece et al., 2013).

Figure 2B shows signaling pathways enriched in diseased liver tissue. The PI3K–Akt pathway was most prominent, driving survival, proliferation, and angiogenesis, while cytokine–cytokine receptor interactions reflected immune activation by macrophages and T cells (Franke et al., 2003; Munn, 2017; Norhalifah et al., 2018; Xue et al., 2021). ECM–receptor and focal adhesion pathways indicated fibroblast-driven remodeling that supports invasion, and enrichment of ribosome-related and immune pathways pointed to increased protein synthesis and systemic immune dysregulation (Munn, 2017; Norhalifah et al., 2018). Together, these results outline a network of proliferative, fibrotic, inflammatory, and angiogenic programs in HCC (Hora and Wuestefeld, 2023; Ma et al., 2025).

Figure 2C highlights increased chemotaxis, consistent with immune recruitment, and enhanced ECM organization from fibroblast activity, along with altered cell adhesion that facilitates tumor spread (Dhar et al., 2020). Elevated translation and ribosome activity reflected the biosynthetic needs of malignant and stromal cells (Scagliola et al., 2023).

Structural changes included collagen-rich ECM remodeling, focal adhesion formation, and basement membrane alterations that enable angiogenesis and tumor migration (Figure 2D). Increased ribosomes and endoplasmic reticulum further emphasized the high protein synthesis required for tumor progression (Yilmaz and Christofori, 2009; Malhi and Kaufman, 2011).

Figure 2E highlights molecular functions enriched in diseased liver tissue, with a strong emphasis on ECM remodeling. Upregulation of structural components, such as collagen, reflects the activity of fibroblasts and stellate cells. In contrast, increased integrin binding and adhesion molecule activity support the greater motility and invasive potential of tumor and stromal cells (Li et al., 2020). Enrichment of heparin and glycosaminoglycan binding indicates modulation of growth factor availability and cytokine signaling, sustaining angiogenesis and inflammation (Taylor and Gallo, 2006). Finally, elevated platelet-derived growth factor (PDGF) binding underscores persistent fibroblast activation and their central role in ECM production and remodeling of the tumor microenvironment (Bonner, 2004).

These findings illustrate the profound cellular and molecular reprogramming that drives HCC progression. The loss of hepatocytes, alongside the expansion of fibroblasts, macrophages, and immune cells, reflects both structural disintegration and immune dysregulation in the liver microenvironment. Enrichment of pathways such as PI3K–Akt, ECM–receptor interactions, and cytokine signaling underscores the interplay of proliferation, fibrosis, inflammation, and angiogenesis, while processes like cell adhesion, ECM biosynthesis, and chemotaxis highlight stromal and immune contributions to a pro-tumorigenic niche. At the molecular level, PDGF signaling, integrin-mediated communication, and matrix remodeling emerge as key mechanisms of invasion and metastasis, offering translational opportunities for therapies targeting fibrotic, immune, and angiogenic axes of HCC.

### 3.4 Cross-cancer validation of biomarkers and pan-cancer expression patterns

To broaden relevance, a pan-cancer analysis was performed across bladder, breast, colon, lung, ovarian, pancreatic, and gastric tumors. This comparison distinguished liver-specific from shared oncogenic biomarkers, providing insight into their potential as diagnostic markers or therapeutic targets beyond HCC. Through integrated analyses of clustering, differential expression, and cross-tumor validation, the study builds a functional landscape that situates liver biomarkers within wider oncogenic networks.

Figures 3A,B display the clustering and expression profiles of key genes across eight cancer datasets, highlighting both the distinctiveness of liver cancer and the pathways it shares with other malignancies. In Figure 3A, HCC forms a separate cluster enriched in genes related to apoptosis resistance, fibrosis, and vascular remodeling, hallmarks of its progression (Baglieri et al., 2019). These liver-specific patterns reflect the impact of chronic injury, metabolic reprogramming, and persistent inflammation, distinguishing HCC from pancreatic, gastric, and lung cancers. While pathways such as ECM remodeling and immune modulation appear across multiple tumors, their expression in non-hepatic cancers lacks the tissue-specific context that characterizes HCC (Pickup et al., 2014).

Figure 3B shows that liver-specific clusters exhibit markedly higher expression of genes related to metabolism and fibrosis in HCC compared to other cancers, where these genes are minimally expressed, supporting their value as diagnostic biomarkers. In contrast, genes regulating angiogenesis and ECM degradation were also expressed in gastric and pancreatic cancers, underscoring the shared tumorigenic pathways (Eble and Niland, 2019). This highlights the dual challenge of identifying truly liver-specific markers while recognizing the convergence of oncogenic mechanisms across malignancies.

To expand this analysis, Figure 3C presents a heatmap of gene expression across bladder, breast, colon, lung, ovary, pancreas, and stomach cancers. Several markers, including Metastasis-Associated Lung Adenocarcinoma Transcript 1 (MALAT1) (a metastasis-promoting lncRNA) and mitochondrial genes Mitochondrially Encoded NADH Dehydrogenase 3 (MT-ND3) and Mitochondrially Encoded NADH Dehydrogenase 2 (MT-ND2), were widely expressed, reflecting common tumor strategies such as angiogenesis, immune modulation (Goyal et al., 2021), and metabolic adaptation through oxidative phosphorylation (Mao et al., 2019; Xu et al., 2023). Other genes of interest include S100 Calcium-Binding Protein A6 (S100A6) and S100 Calcium-Binding Protein A11 (S100A11), both linked to stress responses, epithelial-to-mesenchymal transition (EMT), and increased motility, as well as Mitochondrially Encoded Cytochrome B (MT-CYB), which supports cancer-related metabolic reprogramming through its role in the electron transport chain (Cross et al., 2005; Melle et al., 2008; Yi et al., 2025). Ribosomal and metabolic genes such as Glyceraldehyde-3-Phosphate Dehydrogenase (GAPDH), Ribosomal Protein L41 (RPL41), and Ribosomal Protein S29 (RPS29) also showed strong expression, consistent with the biosynthetic demands of rapidly proliferating tumors (Mao-De and Jing, 2007; Das et al., 2016; Molavi et al., 2019).

Notably, while VCX2 was detected in the dataset, its expression was lower compared to dominant markers such as TMBIM4 and RGS5. Nevertheless, the functional importance of TMBIM4 in apoptosis inhibition and RGS5 in vascular remodeling reinforces their roles in promoting tumor survival and establishing permissive microenvironments for metastatic progression (Silini et al., 2012; Liu, 2017).

Figure 3D presents a tree plot summarizing biomarker enrichment across the dataset, emphasizing pathways and processes linked to tumor growth. Enriched categories included DNA catabolism, nucleocytoplasmic transport, ncRNA processing, ribosome biogenesis, Ras GTPase signaling, and immune regulation, representing core mechanisms of tumor proliferation and genomic instability. Markers such as TMBIM4, RGS5, and MALAT1 were central to processes regulating apoptosis, vascular remodeling, and immune modulation, while ribosomal genes (RPL41, RPS29) reinforced the increased protein synthesis required by cancer cells (Michalik et al., 2014). This hierarchical representation was included to integrate the functional annotations of all candidate biomarkers into a unified view, highlighting the convergence of dysregulated genes toward oncogenic pathways and clarifying how these molecular processes support the prioritization of VCX2 as a key functional target.

The visualization encodes statistical significance by color intensity and pathway size by dot area, highlighting the relative weight of each process. Pathways involving DNA catabolism and nucleocytoplasmic transport involved a large number of genes, underscoring their extensive role in driving genomic instability and tumor growth (Talens and Van Vugt, 2019). Immune regulation pathways, influenced by CEACAM7 and MALAT1, reflected the balance between immune activation, evasion, and tumor-promoting inflammation (Li et al., 2023). Meanwhile, RGS5-associated Ras GTPase signaling pointed to angiogenesis and metastasis (Crosas-Molist et al., 2022).

Overall, the tree plot provides a clear and statistically robust overview of transcriptional reprogramming across cancers. It highlights both shared mechanisms—such as ribosome biogenesis and immune modulation—and context-specific pathways, underscoring the complexity of transcriptional networks that collectively sustain cancer progression (Lallemant et al., 2009; Wang et al., 2010; Ala and Fagoonee, 2024).

Building on this analysis, Figures 4A–G compare differentially expressed genes (DEGs) in HCC with those in seven other cancer types, underscoring the molecular distinctiveness of liver cancer. In Figure 4A, Mitochondrial 2-Like 1 (MTRNR2L1) is upregulated in HCC, reflecting mitochondrial stress responses, while Apolipoprotein A2 (APOA2) highlights liver-specific lipid metabolism reprogramming (Zaki et al., 2013; Rochette et al., 2020). Conversely, the marked downregulation of Albumin (ALB) compared to bladder cancer indicates impaired synthetic capacity in diseased liver tissue (Fu et al., 2022). An immune-related marker, Immunoglobulin Heavy Chain Protein (IGHGP), further emphasizes the contribution of immune responses to HCC pathogenesis (Dunn-Walters et al., 2018). In Figure 4B, the comparison with breast cancer shows elevated X-Inactive Specific Transcript (XIST) expression in breast tumors, reflecting epigenetic regulatory mechanisms, while HCC maintains higher levels of APOA2 and IGHGP, reinforcing its unique reliance on metabolic reprogramming and immune modulation (Vincent-Salomon et al., 2007).

In Figure 4C, the comparison between liver and colon cancers shows higher expression of Trefoil Factors (TFF1 and TFF2) in colon cancer, consistent with their roles in epithelial maintenance and mucosal integrity (Hoffmann, 2005). In contrast, liver cancer exhibits elevated ALB, APOA2, and Immunoglobulin Heavy Constant Gamma 3 (IGHG3), highlighting liver-specific metabolic alterations and B-cell–mediated immune responses (Zhao J. et al., 2021). Figure 4D contrasts liver and lung cancers. Lung tumors display high levels of Eukaryotic Translation Initiation Factor 1A, Y-linked (EIF1AY) (Fan et al., 2014), reflecting enhanced protein synthesis, while HCC shows pronounced expression of fibrinogen components (FGA, FGB, FGG) and Transthyretin (TTR), pointing to hypercoagulability and disrupted metabolic regulation (Kenarangi et al., 2022).

The role of regulatory genes in HCC progression is reinforced by markers such as RGS5, which is strongly expressed in endothelial cells and macrophages, where it promotes vascular remodeling and neovascularization (Kong et al., 2023). Although VCX2 is less abundant than TMBIM4 or RGS5, its distinct profile in diseased liver tissue suggests a role in chromosomal stability and tumor proliferation, potentially acting synergistically with other markers (Cuevas-Covarrubias and González-Huerta, 2008). Additionally, CEACAM7, expressed in immune cells, contributes to inflammation and immune evasion, further supporting a pro-tumorigenic environment (Kelleher et al., 2019).

Figure 4E compares liver and ovarian cancers, highlighting elevated glutathione peroxidase 1 (GPX1) in HCC (Lei et al., 2007), a regulator of oxidative stress, alongside APOA2 and IGHGP, which reinforce its distinct metabolic and immunological profiles. In contrast, ovarian cancer shows high expression of XIST, reflecting differences in epigenetic regulation.

In Figure 4F, liver and pancreatic cancers are contrasted. HCC shows higher expression of ATP Synthase Subunit E (ATP5E) and Guanine Nucleotide-Binding Protein Subunit Beta-2-Like 1 (GNB2L1), genes tied to mitochondrial bioenergetics and metabolic reprogramming (Huang et al., 2019), while pancreatic cancer relies more on Osteopontin (SPP1), associated with ECM remodeling and metastasis, though it is also moderately elevated in HCC (Nallasamy et al., 2021). Figure 4G shows that stomach cancer is enriched in TFF1 and TFF2, linked to mucosal defense, whereas HCC exhibits higher ALB, APOA2, IGHG1, and fibrinogen components (FGA, FGB, FGG), consistent with inflammation and a pro-thrombotic microenvironment.

Figure 4H examines the biomarker distribution across different cell types. TMBIM4 is broadly expressed, underscoring its role in apoptosis inhibition and stress resilience (Liu, 2017). While VCX2 is confined mainly to gametocytes and hepatocytes, suggesting a specialized function in chromosomal stability and liver-specific processes (Jakobsen et al., 2021). RGS5 shows high expression in osteoblasts, smooth muscle, and stem cells, reflecting its role in vascular remodeling and angiogenesis (Arnold et al., 2014).

The restricted expression of VCX2, a CTA, in hepatocytes has significant implications (Jakobsen et al., 2021; Yin et al., 2024). According to Zhang et al. (2023), which usually remains silent in somatic tissues due to DNA methylation, VCX2 can be reactivated in cancer through epigenetic dysregulation, thereby supporting immune evasion and intratumoral heterogeneity. This plasticity suggests potential for VCX2 as both a biomarker and a therapeutic target for epigenetic and immune-based interventions.

Additional genes of note include Keratin 17 (KRT17), linked to epithelial integrity and invasiveness (Zhang et al., 2022); Keto Reductase Family 1 Member C3 (AKR1C3), associated with steroid metabolism and differentiation (Li et al., 2024); and Endothelial cell-specific chemotaxis receptor (ECSCR), reinforcing endothelial signaling and angiogenesis (Verma et al., 2010). Although overshadowed by dominant markers like TMBIM4 and RGS5, low-abundance transcripts such as Ataxin 8 Opposite Strand (ATXN8OS) (Deng et al., 2019) and RP11-661A12.4 may also play roles in transcriptional regulation and warrant further study.

In summary, Figure 4H highlights the distinct expression patterns of key biomarkers across cell types. TMBIM4 shows broad distribution across nearly all lineages, underscoring its universal role in apoptosis regulation and stress resilience. In contrast, markers such as VCX2, RGS5, KRT17, AKR1C3, and ECSCR display restricted, cell-type–specific expression, suggesting specialized functions (Verma et al., 2010). Notably, the enrichment of VCX2 in hepatocytes reinforces its potential as a liver-specific marker in HCC, linking it to chromosomal stability and tumor adaptation. The detection of low-abundance transcripts such as ATXN8OS and RP11-661A12.4 further points to the importance of exploring less prominent markers that may hold significant regulatory roles.

Figure 4I extends this analysis by evaluating cross-cancer gene expression. Expression is quantified by average intensity (color gradients) and the percentage of cells expressing each marker (dot size). VCX2 emerges with pronounced, liver-specific expression, particularly in HCC, underscoring its potential in chromosomal regulation, immune evasion, and tumor progression. Given its CTA-like expression profile, VCX2 may represent a conceptual framework for future immunotherapeutic exploration, pending confirmation of protein-level tumor specificity. In contrast, TMBIM4 maintains broad expression across organs and cancers, confirming its function as a universal regulator of apoptosis and stress response (Carrara et al., 2017).

Within the liver, VCX2, RGS5, RP11-661A12.4, and ATXN8OS stand out as significant biomarkers. VCX2 shows enriched expression in HCC relative to other tumor datasets, while RGS5 contributes to angiogenesis and vascular remodeling (Hamzah et al., 2008). The lncRNAs RP11-661A12.4 and ATXN8OS exhibit liver-restricted expression, indicating potential regulatory roles in metabolic reprogramming and oncogenesis. Together, these markers highlight the interplay between metabolic, vascular, and epigenetic mechanisms in shaping liver cancer biology and reinforce their potential as diagnostic and therapeutic targets (Roh et al., 2022).

Figure 4J examines the combinatorial expression of VCX2, ATXN8OS, and RP11-661A12.4 to identify synergistic interactions driving HCC progression. The strongest effect was observed for VCX2 + ATXN8OS, where VCX2’s role in chromosomal stability appears to enhance the oncogenic transcriptional activity of ATXN8OS (Kumar et al., 2020; Prabhakaran et al., 2024). The VCX2 + RP11-661A12.4 pair also showed synergy, likely through integration of genomic regulation and transcriptional control, whereas the ATXN8OS + RP11-661A12.4 combination was comparatively weaker. Interestingly, while the complete triad remained highly expressed in pathological tissue, it did not exceed the VCX2 + ATXN8OS pair, underscoring the central influence of VCX2 in liver tumorigenesis. VCX2 as a potential regulatory node and candidate for future immunotherapeutic exploration in HCC.

Building on these findings, subsequent analyses reinforced VCX2’s involvement in chromosomal regulation, transcriptional networks, and tumor progression, supporting its candidacy for targeted therapies. The broader synthesis of Figure 4 emphasizes how transcriptional reprogramming and cellular restructuring—characterized by hepatocyte loss and the expansion of fibroblasts, macrophages, and immune cells—contribute to the pathological microenvironment of HCC. Within this context, TMBIM4 and RGS5 emerge as complementary regulators, promoting survival, angiogenesis, and immune evasion. Together, these biomarkers highlight key oncogenic mechanisms and therapeutic vulnerabilities in liver cancer.

To explore VCX2’s broader functional role, we examined its integration within gene and protein interaction networks (Figure 5). The upper panel displays three network diagrams, with Figure 5A illustrating a node-link structure derived from human liver expression data. Here, VCX2 (light pink) is positioned as a central hub connected to multiple interacting genes and proteins (blue and gray nodes). These associations indicate links to chromatin organization, immune modulation, and transcriptional regulation, underscoring VCX2’s potential to coordinate chromosomal stability with liver-specific oncogenic signaling. The clustering pattern within the network highlights its role as an integrative regulator in HCC biology.

Figure 5B further establishes VCX2 as a central hub within the interaction network, represented as a red node with the highest centrality scores, underscoring its dominant regulatory potential. VCX2 directly connects to functionally significant genes, including Steroid Sulfatase (STS), Arylsulfatase F (ARSF), Pseudouridine 5′-Phosphatase (PUDP), and Patatin-Like Phospholipase Domain Containing 4 (PNPLA4) (orange/yellow nodes). These interactors span key processes relevant to tumor biology: STS in steroid metabolism and hormone regulation (Reed et al., 2005), ARSF in glycosaminoglycan metabolism and ECM integrity (Feferman et al., 2013), PUDP in RNA modification and stability (Yu et al., 2022), and Patatin-Like Phospholipase Domain Containing 4 (PNPLA4) in lipid metabolism, particularly relevant in liver cancer (Björnson et al., 2015). The size and prominence of these nodes highlight the strength of these associations and reinforce VCX2’s role in integrating diverse regulatory pathways in HCC.

The interaction network in Figure 5C highlights the complexity of VCX2’s molecular associations, linking it to diverse processes including chromosomal regulation, transcriptional control, lipid metabolism, and tumor progression (Jakobsen et al., 2021). Color gradients and segmented nodes denote overlapping functions across datasets, while interactions with Heat Shock Transcription Factor Family, X-linked 2 (HSFX2), and Melanoma-Associated Antigen 10 (MAGEA10) connect VCX2 to other CTAs implicated in immune evasion and proliferation (Zendman et al., 2002). These associations reinforce VCX2’s potential functional relevance but also raise the need for structural validation to ensure that downstream modeling and screening efforts rely on a biologically meaningful conformation.

To better integrate the transcriptomic and structural findings, we propose a systems-level hypothesis linking VCX2 expression patterns with its structural properties. As a cancer/testis-like gene with context-dependent expression in hepatocellular carcinoma, VCX2 may function as a conformationally dynamic regulatory protein rather than a classical enzyme. The presence of partially disordered regions and the formation of metastable, solvent-exposed binding grooves suggest that its functional role may involve transient protein–protein interactions or participation in regulatory complexes within tumor-associated cellular states.

Within this framework, ligand binding is not expected to induce classical inhibition, but rather to stabilize local conformational states within the VCX2 structure. This stabilization may reduce conformational plasticity and perturb transient interaction interfaces, thereby modulating VCX2-associated regulatory processes. Although this hypothesis requires experimental validation, it provides a mechanistic bridge between transcriptomic prioritization, structural druggability, and ligand interaction.

### 3.5 Structural modeling and molecular dynamics refinement of VCX2

To strengthen the structural validation of VCX2, we performed a comparative modeling analysis across multiple prediction frameworks prior to molecular dynamics refinement (Supplementary Figure S1). The AlphaFold-predicted model (139 amino acids) exhibited an elongated, low-confidence conformation (average pLDDT = 62.6, with ∼89% of residues < 70), suggesting a largely disordered architecture. PSIPRED/DISOPRED3 analyses confirmed three α-helices (residues 43–49, 60–77, 118–130) embedded within highly flexible coil regions (disorder probability > 0.9).

Threading-based modeling using Phyre2 failed to identify reliable structural homologs (confidence < 20%), and meta-threading via I-TASSER produced only low-confidence models (top C-score = −3.37; estimated TM-score = 0.34 ± 0.11) derived from low-identity, coil-rich templates (Iden1 ≤ 0.13). Its secondary-structure prediction classified nearly the entire sequence as coil, supporting the intrinsically disordered character of VCX2. Independent AI predictors (Chai-1: aggregate 0.028, pTM 0.139; Boltz-2: confidence 0.463, pTM 0.218, pLDDT 0.524) similarly yielded unstructured, extended topologies. Together, these results indicated that VCX2 likely exists as a partially disordered protein capable of forming localized helical elements upon stabilization.

To evaluate the structural fidelity of VCX2, we performed molecular dynamics simulations across three independently generated models, followed by PCA. The eigenvalue decay curves (Supplementary Figure S2A, left) revealed consistent variance profiles across replicates, while the PCA projection of the 1,000 ns trajectory (Supplementary Figure S2A, right) identified distinct conformational clusters, indicating that metastable states were sampled during the simulation. Stereochemical validation using Ramachandran plots (Supplementary Figure S2B) showed a substantial improvement in model quality: residues in favored regions increased from 54% in the AlphaFold-predicted model to 89% after refinement, with disallowed conformations eliminated (Alhumaid and Tawfik, 2024; Baselious et al., 2024; Lyu et al., 2024). Global structural evaluation with ProSA-web further supported this refinement, as the Z-score of the optimized model fell within the range of experimentally solved X-ray structures, and local energy distributions were markedly improved (Supplementary Figure S2C). Finally, structural comparison of the initial and refined models (Supplementary Figure S2D) demonstrated increased compactness, improved secondary structure definition, and reduced conformational disorder, validating the suitability of the refined VCX2 structure for downstream virtual screening.

Electrostatic mapping of the MD-refined VCX2 model (Supplementary Figure S3) revealed a clear bipolar charge distribution, with a Lys/Arg-enriched N-terminal region (residues 15–60) and an Asp/Glu-enriched C-terminal region (residues 100–131) located on opposing surfaces of the protein. The highest-scoring SiteMap pocket—including residues Ala61, Thr63, and Glu68—lies within this negatively charged surface region, which remained stable throughout the 1 μs trajectory. This electrostatic landscape provides additional structural support for selecting this site as the primary binding region for subsequent docking and screening analyses.

Building on this validated model, the three-dimensional representation in Figure 5D highlights distinct ligand-binding pockets (blue, orange, pink), with the blue pocket exhibiting the highest site score (1.009, Maestro SiteMap) and therefore selected for further analysis. Structural refinement yielded a stable conformation, with RMSD values stabilizing between 0.36 and 0.79 nm and Ramachandran analysis confirming improved geometry (89% residues in favored regions vs. 54% in the initial AlphaFold model) (Supplementary Table S1; Supplementary Figure S2). While high-resolution experimental data will be required to fully validate the predicted binding site, these results support the structural consistency of the identified binding region of the refined VCX2 model (García-Nafría and Tate, 2020).

Importantly, the prioritization of VCX2 as a potential therapeutic target was not based solely on its transcriptomic profile but was further supported by convergent structural evidence. The MD-refined model consistently revealed a solvent-accessible binding groove localized around residues Ala61, Thr63, and Glu68, which persisted throughout the simulation and supported stable ligand accommodation during subsequent docking and molecular dynamics analyses.

This binding region is best interpreted as a metastable, MD-stabilized polar groove formed within a partially ordered segment of an otherwise partially disordered protein, rather than as a rigid pocket embedded in a fully structured domain. Its persistence across long-timescale simulations argues against a purely transient or artifactual cavity, while still acknowledging the dynamic nature of the system and the need for experimental structural validation.

A limitation of the present study is the partially disordered nature of VCX2, which poses inherent challenges for structure-based drug design. Unlike well-folded proteins with stable binding pockets, intrinsically disordered or highly flexible proteins sample multiple conformational states, complicating the identification of persistent and functionally meaningful ligand-binding sites. In this context, the VCX2 site identified here should not be interpreted as a classical rigid pocket, but rather as a dynamically stabilized groove emerging from the conformational ensemble sampled during molecular dynamics refinement. Although this behavior introduces uncertainty into docking-based predictions, it is consistent with current efforts to target transient or disorder-to-order–prone regions in flexible proteins. Therefore, the present results should be interpreted as an initial structural exploration of ligand accessibility and interaction potential in VCX2, rather than as definitive evidence of conventional druggability.

Accordingly, the concept of druggability in the context of VCX2 should be interpreted with caution. While the MD-refined structure revealed a ligand-accessible region capable of supporting stable interactions, the current evidence is based on a relatively small, biodiversity-focused compound library and computational validation alone. Therefore, these findings do not establish definitive druggability, but rather support the ligandability (pocket-level binding feasibility) of a dynamically stabilized VCX2 binding region and should be considered hypothesis-generating, requiring validation using larger and more diverse chemical libraries as well as experimental approaches.

### 3.6 Virtual screening and docking of Peruvian natural products against VCX2

The PeruNPDB library represents a literature-curated collection of natural products derived from Peruvian biodiversity, capturing a region-specific and still underexplored chemical space. Although relatively small in size (280 compounds), it encompasses diverse chemical classes, including flavonoids, alkaloids, terpenoids, and phenolic compounds, many of which are associated with known bioactivities and ethnopharmacological relevance. As part of emerging Latin American natural product initiatives, PeruNPDB provides a focused, biodiversity-driven framework for the identification of biologically meaningful scaffolds.

Building on this chemical space, virtual screening of the PeruNPDB library was performed to identify potential VCX2-binding compounds, focusing on the top-ranked binding pocket identified by site mapping. Interactions with binding energies weaker than −2 kcal/mol were filtered out. Figure 5E shows the Z-score distribution of binding affinities, highlighting several compounds with favorable predicted interactions. Among these, Luteolin-5-O-glucoside (PeruNPDB277) emerged as the top candidate, falling within the high-affinity region. Figure 5F depicts its chemical structure, classifying it as a flavonoid glycoside commonly found in plant species such as *Cirsium maackii* and *E. arvense* (Jung et al., 2012; Carneiro et al., 2019). Notably, *Equisetum arvense* has been associated with traditional medicinal use, including anti-inflammatory and wound-healing applications, consistent with the reported bioactivity of luteolin-derived compounds (Parameshwaran et al., 2022).

This finding is also biologically plausible in light of prior literature on luteolin-related flavonoids. Although direct evidence for luteolin-5-O-glucoside in hepatocellular carcinoma remains limited, the luteolin scaffold has been associated with anticancer activity in liver cancer–relevant systems, including inhibition of proliferation and induction of apoptosis through modulation of survival-associated pathways such as PI3K/AKT (Park and Song, 2013; Pirvu et al., 2020). Moreover, related flavonoid glycosides have been reported to induce growth inhibition, cell-cycle arrest, and oxidative stress–mediated responses in cancer models (Palko-Labuz et al., 2017). Taken together, these observations support the biological plausibility of luteolin-derived flavonoids as relevant scaffolds in HCC-associated contexts, while still underscoring that the activity of PeruNPDB277 itself remains to be experimentally validated.

To further characterize its inhibitory potential, we examined the molecular basis of Luteolin-5-O-glucoside binding to VCX2 using docking, 200-ns MD refinement, and MM-GBSA. The multiple hydroxyl groups of the ligand enable extensive hydrogen bonding and water-bridge formation within the binding site, consistent with the highly polar nature of the pocket. Luteolin-5-O-glucoside obtained a Glide docking score of −3.949 ± 0.85 kcal/mol. Although modest, this behavior is expected for shallow polar pockets, where scoring functions tend to underpredict affinity. MM-GBSA calculations provided a more accurate assessment, yielding a binding free energy of ΔG_bind_ = −35.43 ± 1.12 kcal/mol, indicative of a stable and thermodynamically favorable interaction.

Importantly, docking scores should not be interpreted as direct measures of binding affinity, particularly in solvent-exposed polar environments where electrostatic and solvent-mediated interactions dominate. In such contexts, empirical scoring functions are known to underpredict binding strength due to their limited treatment of solvation effects and conformational flexibility. Accordingly, the more favorable MM-GBSA binding free energy and the stability observed during molecular dynamics simulations provide stronger evidence supporting the viability of this interaction. Therefore, luteolin-5-O-glucoside should be considered a preliminary lead scaffold suitable for further optimization rather than a fully optimized drug candidate.

To further assess the translational relevance of the top-ranked ligand, basic drug-likeness and ADMET properties were evaluated for PeruNPDB277. The predicted profile (Supplementary Table S2) indicates high polarity (TPSA = 190.28 Å^2^), multiple hydrogen bond donors and acceptors, and low gastrointestinal absorption, leading to violations of several classical drug-likeness criteria and a low predicted bioavailability score (0.17). These features indicate limited suitability as an orally bioavailable drug in its current form. At the same time, PeruNPDB277 showed favorable aqueous solubility, no predicted inhibition of major CYP isoforms, and no P-glycoprotein substrate liability, suggesting a relatively benign early pharmacokinetic and toxicity profile despite its low permeability. Taken together, these results support its interpretation as a preliminary bioactive scaffold rather than a fully optimized drug candidate, and indicate that future optimization or alternative delivery strategies would likely be required to improve translational potential.

Experimental validation will be essential to determine whether this predicted interaction has functional relevance in a biological context. Feasible follow-up approaches include biophysical binding assays, such as thermal shift assays or surface plasmon resonance, to verify ligand–protein interaction, as well as hepatocellular carcinoma cell-based assays to assess effects on cell viability, proliferation, and related phenotypic responses. Given the partially disordered nature of VCX2, such studies will also be important to determine whether ligand binding promotes local structural stabilization or perturbs transient interaction surfaces associated with VCX2-related regulatory functions.

Beyond this initial observation, the discrepancy can be rationalized by considering the physicochemical context of the VCX2 binding surface. In contrast to deeply buried hydrophobic pockets, ligand binding in solvent-exposed polar grooves is largely driven by electrostatic interactions, solvent reorganization, and dynamic conformational adaptation. These contributions are only partially captured by empirical docking scores. By incorporating structural relaxation and solvation effects along the MD trajectory, MM-GBSA provides a more comprehensive representation of binding energetics in this system, supporting a model in which ligand recognition occurs within a dynamically stabilized, hydrogen bond–rich environment.

To characterize the molecular determinants of binding, Figure 6A summarizes the key interactions stabilizing the complex. Prominent polar contacts were formed with Glu68, Thr63, Gln82, Ala60, Ala61, Val62, and Met59, which outline the solvent-exposed groove in which the ligand resides. These residues collectively form a dense network of electrostatic and hydrogen-bond interactions, reinforced by structured water molecules.

To assess the stability of the VCX2–Luteolin-5-O-glucoside complex, we evaluated backbone fluctuations and residue-level energies over a 200-ns MD simulation (Supplementary Figure S4). The complex remained stable, with RMSD values plateauing at 0.6–0.8 nm after ∼60 ns (Supplementary Figure S4A), consistent with expected relaxation in partially disordered proteins. RMSF analysis showed low flexibility within the binding region (residues 59–70; <0.35 nm), indicating stable ligand accommodation (Supplementary Figure S4B).

Per-residue energy decomposition (Supplementary Figure S4C) indicated that the interaction is supported by both electrostatic and van der Waals contributions, with Glu68, Gln82, Ala61, Val62, and Met59 providing the strongest stabilizing effects. This profile is consistent with a binding groove environment in which polar interactions and surface complementarity jointly contribute to molecular recognition. These results demonstrate sustained complex stability and support the reliability of the predicted binding mode.

To rigorously assess the stability of the identified binding pocket, a quantitative residue-level interaction analysis was performed across the MD trajectory. Hydrogen bond analysis revealed that residues Glu68 (191 events), Thr63 (154 events), and Ala60 (153 events) were among the most frequent contributors, indicating sustained polar interactions within the binding region.

Additionally, water-mediated interactions were highly prevalent, with Ser69 (319 events), Glu68 (295 events), and Thr63 (225 events) showing strong participation, highlighting the role of solvent-mediated stabilization in this predominantly hydrophilic pocket. Hydrophobic contacts were less prominent but consistently observed for residues such as Ala67, Val62, and Ala64, suggesting a secondary structural contribution.

Importantly, these interactions were concentrated within a contiguous region spanning residues Ala60–Glu68, forming a coherent interaction network that persisted throughout the simulation. Together, these findings demonstrate that the selected SiteMap pocket corresponds to a dynamically stable and structurally consistent binding region rather than a transient conformational artifact.

MD simulations were performed to evaluate the dynamic stability of the ligand within the refined VCX2 pocket. MM-GBSA component analysis (Supplementary Table S3) showed that binding is supported by both electrostatic and van der Waals contributions, with favorable Coulomb (−24.79 kcal/mol) and vdW (−28.10 kcal/mol) terms, together with additional hydrogen-bond contributions (−3.72 kcal/mol). These stabilizing interactions are partially offset by the positive solvation contribution (Solv GB = +26.83 kcal/mol), consistent with ligand recognition in a shallow, solvent-exposed, and hydrophilic groove rather than in a deeply buried hydrophobic pocket. The most stabilizing residues were Glu68 (−50.81 kcal/mol) and Gln82 (−47.51 kcal/mol), followed by Ala61 (−24.77 kcal/mol), Val62 (−23.02 kcal/mol), Met59 (−22.12 kcal/mol), and Ala60 (−19.51 kcal/mol), while Thr63 (−5.32 kcal/mol) contributed additional transient hydrogen bonds. Together, these results indicate that ligand recognition in this region is governed by mixed polar and surface-complementarity interactions, rather than by hydrophobic packing alone (Figure 6B).

Overall, the identification of Luteolin-5-O-glucoside as a stable VCX2-binding ligand marks a preliminary but meaningful step in exploring the druggability of this understudied cancer/testis antigen. The ligand’s stable MD profile, favorable MM-GBSA energy, and consistent interaction pattern support its potential as a lead scaffold for future optimization. As no experimentally validated VCX2 inhibitors have been reported to date, direct benchmarking against reference compounds was not feasible. Furthermore, due to the absence of known active or inactive ligand sets for VCX2, conventional redocking and decoy-docking validation strategies could not be applied in this system. Therefore, ligand prioritization relied on internal ranking criteria, including GlideScore, Z-score normalization, MM-GBSA binding free energy estimates, per-residue interaction analysis, and long-timescale molecular dynamics stability analysis. The convergence of these orthogonal computational metrics strengthens confidence in the identified candidate and provides a foundation for future structure–activity relationship (SAR) studies and experimental validation. Future studies incorporating decoy sets or larger comparator libraries will be valuable to further assess pose discrimination and binding selectivity.

Within this context, although PeruNPDB is smaller than large public compound repositories typically used for high-throughput virtual screening, it was intentionally selected as a curated, biodiversity-focused library enriched in Peruvian natural products. Accordingly, the present screening should be interpreted as a hypothesis-generating exploration of region-specific chemical space rather than as an exhaustive assessment of pocket selectivity. Future studies incorporating larger and more chemically diverse libraries will be important to further evaluate ligand selectivity for the VCX2 binding site.

Given the absence of a well-defined catalytic domain and the partially disordered nature of VCX2, the functional impact of ligand binding is unlikely to involve classical active-site inhibition. Instead, binding of luteolin-5-O-glucoside is more plausibly interpreted as a modulatory event that locally stabilizes the Ala61–Glu68 region, reducing conformational plasticity and altering the dynamic properties of the binding surface. Such stabilization may perturb transient interaction interfaces or conformational selection processes associated with VCX2 regulatory function. While this hypothesis requires experimental validation, it provides a mechanistic framework consistent with the structural and dynamic features observed in this study.

The detection of VCX2 transcripts in a subset of hepatocytes also warrants caution when considering translational relevance. Although VCX2 shows a cancer/testis antigen–like profile, low-level expression in non-malignant liver cells could potentially narrow the therapeutic window and raise the possibility of on-target hepatotoxicity. At the same time, transcript detection by scRNA-seq does not necessarily imply equivalent protein abundance, functional activity, or pharmacological accessibility in normal hepatocytes. Therefore, the therapeutic implications of VCX2 expression in liver tissue should be interpreted cautiously and validated experimentally through protein-level characterization in normal and tumor samples, together with hepatocyte-based selectivity and toxicity assays. These considerations will be particularly important for assessing the feasibility of future VCX2-targeted therapeutic strategies.

It is important to note that the transcriptomic analyses presented here integrate independently generated healthy and tumor scRNA-seq datasets. Although normalization and statistical controls were applied to minimize technical variability, residual batch effects cannot be completely excluded. Therefore, the disease-associated expression pattern of VCX2 should be interpreted as a hypothesis-generating observation that warrants validation in independent, clinically matched cohorts generated under uniform sequencing conditions.

## 4 Conclusion

This study was designed to identify and prioritize candidate molecular targets in HCC through an integrative computational framework, leading to the selection of VCX2 for in-depth structural and druggability assessment. Transcriptomic analyses, including scRNA-seq, revealed differential expression of VCX2 in HCC compared to healthy liver tissue. Pan-cancer comparisons across bladder, breast, colon, lung, ovarian, pancreatic, and stomach cancers support the context-dependent expression pattern of VCX2, suggesting liver-associated relevance rather than universal oncogenic behavior, with potential associations with tumor progression and chromosomal regulatory processes.

Following its biological prioritization, we performed a comprehensive *in silico* characterization of VCX2 to evaluate its structural druggability. Comparative modeling using AlphaFold, PSIPRED, Phyre2, I-TASSER, Chai-1, and Boltz-2 established VCX2 as a partially disordered protein containing stable helical subdomains. Long-timescale molecular dynamics (1,000 ns) refinement generated a compact and stereochemically validated structure, enabling reliable identification of druggable surfaces. Site-mapping revealed multiple cavities, with the highest-scoring pocket (SiteMap score = 1.009) selected for virtual screening.

Screening of the PeruNPDB database identified Luteolin-5-O-glucoside (PeruNPDB277)—a flavonoid glycoside present in *E. arvense*—as the top VCX2-binding candidate. The ligand achieved a Glide docking score of −3.949 ± 0.85 kcal/mol and an MM-GBSA binding free energy of ΔG_bind_ = −35.43 ± 1.12 kcal/mol, dominated by strong electrostatic contributions. The MD-refined binding mode revealed a network of persistent hydrogen bonds and polar contacts with Glu68, Thr63, Gln82, Ala60, Ala61, Val62, and Met59, consistent with the highly polar nature of the pocket. A 200 ns MD simulation further confirmed the stability of the complex, with RMSD and RMSF analyses indicating sustained ligand accommodation.

These findings support the computational prioritization of VCX2 as a structurally druggable candidate in HCC and identify Luteolin-5-O-glucoside as an initial scaffold for further exploration. Because the VCX2 structure remains predictive, future work should include experimental structural determination (X-ray crystallography or cryo-EM) alongside *in vitro* and *in vivo* validation to confirm its therapeutic potential.

This study demonstrates how an integrative omics-guided computational pipeline—combining single-cell transcriptomics, pan-cancer profiling, structural modeling, and virtual screening—can prioritize previously underexplored molecular candidates such as VCX2 in HCC. The identification of Luteolin-5-O-glucoside as a stable VCX2-binding ligand provides an initial proof-of-concept for structure-based exploration of VCX2-associated pathways and supports its consideration as a preliminary bioactive scaffold.

From a translational perspective, however, these findings should be interpreted within the context of early-stage computational discovery. While luteolin-5-O-glucoside (PeruNPDB277) demonstrates favorable binding energetics and dynamic stability *in silico*, its physicochemical profile—characterized by high polarity and low predicted gastrointestinal absorption—suggests limited oral bioavailability in its current form. This highlights the need for further optimization through structure–activity relationship (SAR) strategies and/or alternative delivery approaches to improve pharmacokinetic properties.

Experimental validation will be essential to confirm the biological relevance of the predicted interaction. Feasible approaches include biophysical binding assays (e.g., thermal shift assays or surface plasmon resonance) and hepatocellular carcinoma cell-based studies to assess functional effects. Given the partially disordered nature of VCX2, such studies will be particularly important to determine whether ligand binding induces local structural stabilization or perturbs transient interaction interfaces associated with its regulatory role.

## Data Availability

The software used in this study includes Maestro (Schrödinger Suite), Desmond, SiteMap, and Glide, all of which require a paid license for both academic and commercial use. Additionally, STRING, Cytoscape, and PASSer were utilized, which are freely available for non-commercial use. All software tools are referenced throughout the Methods and Results sections. Furthermore, protein structures, docking grids, molecular dynamics trajectories, ligand preparation files, energy minimization data, and analysis scripts generated during this study are documented accordingly. All simulation scripts, docking results, molecular interaction analyses, and processed datasets can be found at https://figshare.com/s/d13d8bcd01fbf5487038 and https://github.com/CompBioChemRG/SingleCell_CBCRG.
