# Supplementary material for: An integrative omics-guided druggability analysis of VCX2 in hepatocellular carcinoma using Peruvian natural products
Source: Front Bioinform. 2026 May 18;6:1822441. doi: 10.3389/fbinf.2026.1822441 (PMC13222996; doi:10.3389/fbinf.2026.1822441)
Supplement: Supplementary file 1 [file Supplementaryfile1.docx]

Supplementary Material

**An Integrative Omics-Guided Druggability Analysis of VCX2 in Hepatocellular Carcinoma Using Peruvian Natural Products**

Luis Daniel Goyzueta-Mamani^1^, Haruna Luz Barazorda-Ccahuana^1^, Mayron Antonio Candia-Puma^1,2^, Nadia M. Hamdy^3^ and Miguel Angel Chávez-Fumagalli^1^*

## Supplementary Figures


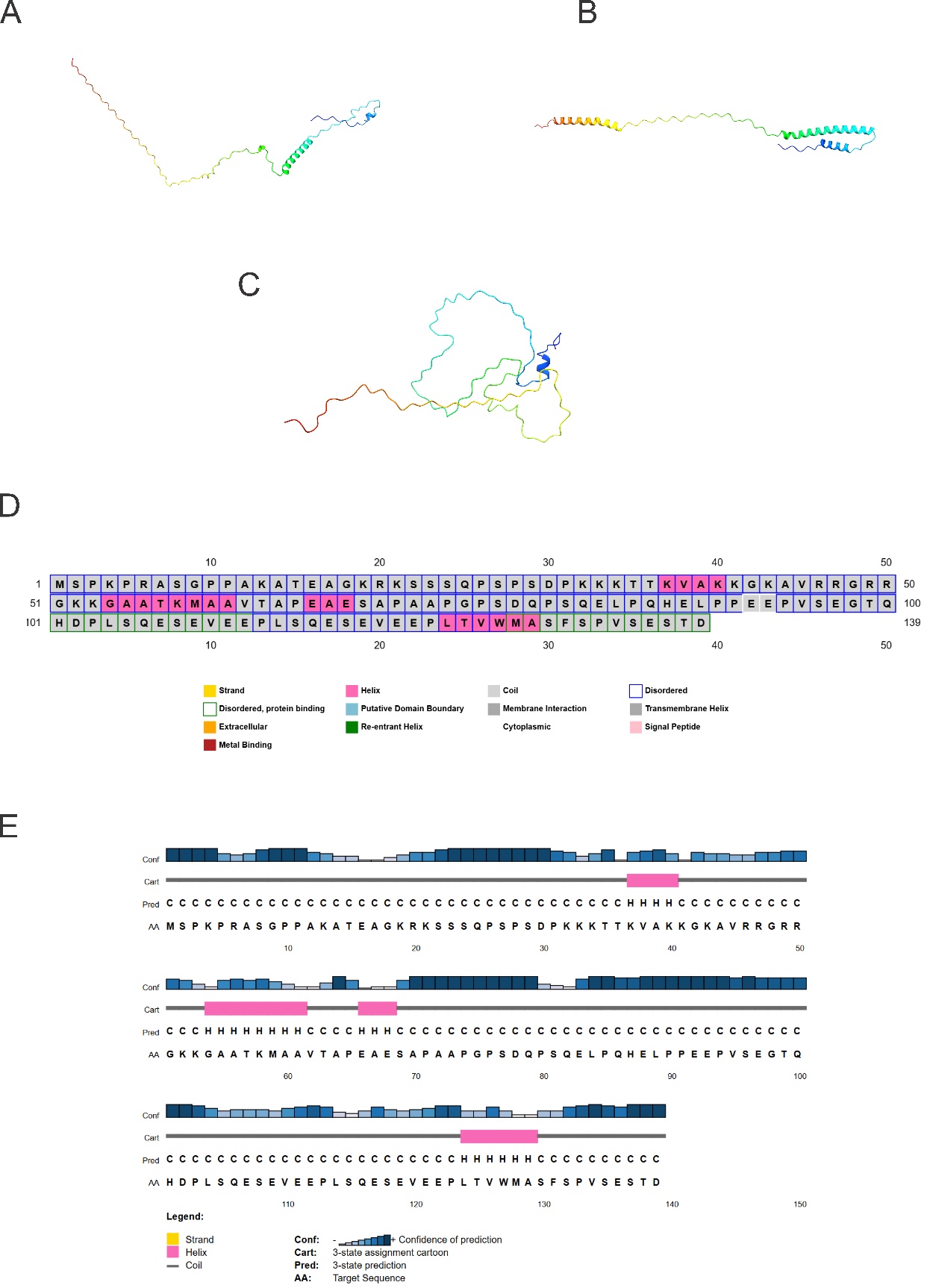


**Figure S1.** Comparative AI-based and secondary-structure predictions for VCX2. **(A)** Predicted tertiary structure generated by Chai-1, **(B)** Model from Boltz-2, **(C)** Top-ranked I-TASSER model, **(D)** Predicted secondary-structure composition from PSIPRED/ProMotif, and **(E)** Confidence map from PSIPRED.


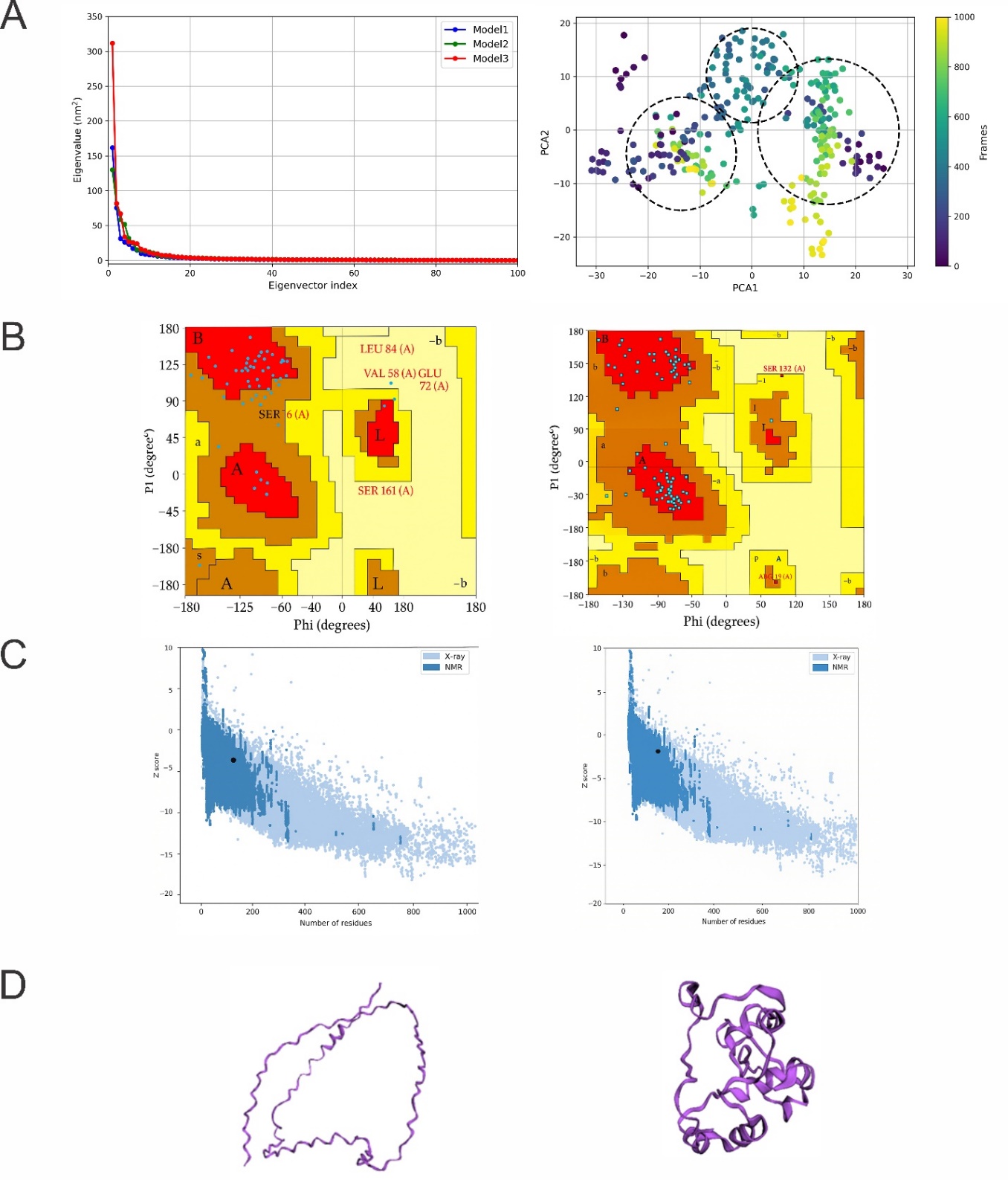


**Figure S2.** Principal component analysis (PCA) of protein dynamics. **(A)** (left) Eigenvalue plots for three independently modeled protein structures (Model1–Model3) show consistent decay, indicating stable and convergent dynamics. (right) PCA projection of a 1000 ns trajectory, with frames colored by time. Dashed circles highlight distinct conformational clusters, suggesting the presence of metastable states sampled during the simulation. Structural validation of the VCX2 (Variable Charge, X-linked 2) protein model before (left column) and after (right column) refinement. **(B)** Ramachandran plots (PROCHECK) show increased occupancy in favored regions post-refinement. **(C)** ProSA-web Z-scores indicate enhanced model quality compared to reference X-ray and NMR structures. **(D)** 3D cartoon representations of VCX2 show improved folding and compactness model.


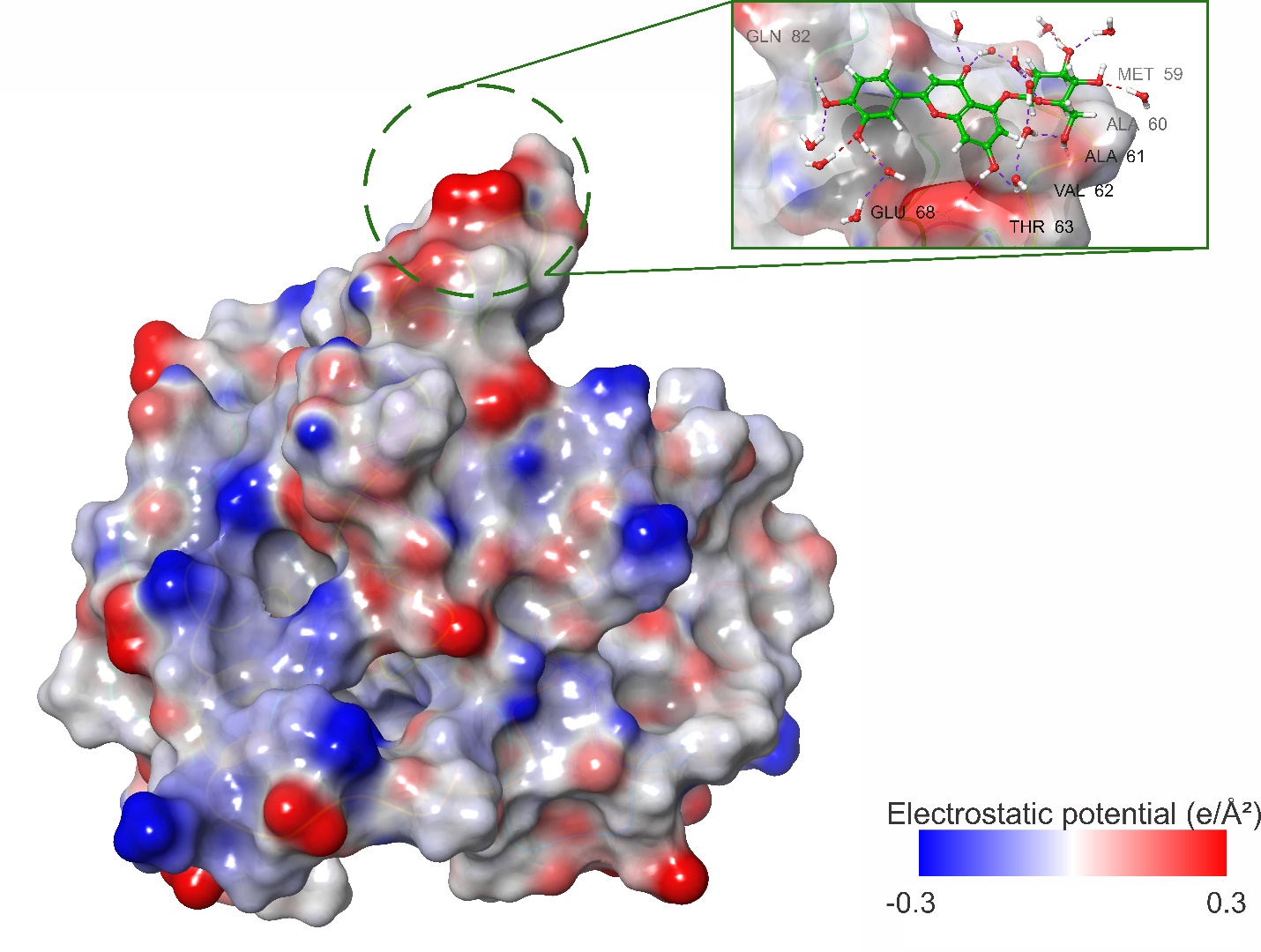


**Figure S3.** Electrostatic surface potential of the MD-refined VCX2 structure. The electrostatic potential surface was calculated in Schrödinger Maestro v12.8 using the OPLS4 force field at pH 7.4.


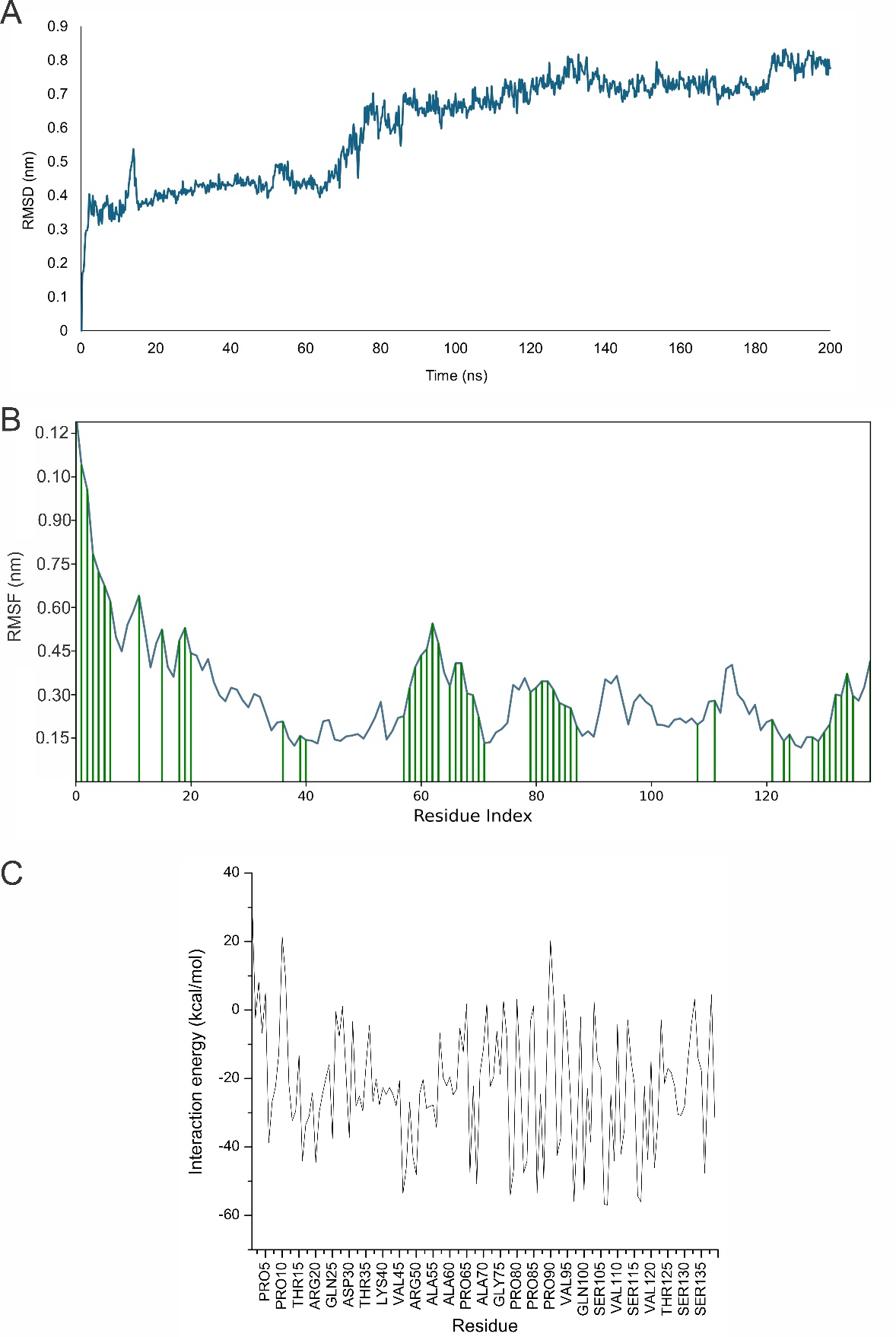


**Figure S4.** MD-derived stability and interaction profile of the VCX2–Luteolin-5-O-glucoside complex. **(A)** RMSD over 200 ns; **(B)** RMSF per residue; **(C)** MM-GBSA per-residue interaction energies identifying Glu68, Gln82, Ala61, Val62, and Met59 as dominant contributors.


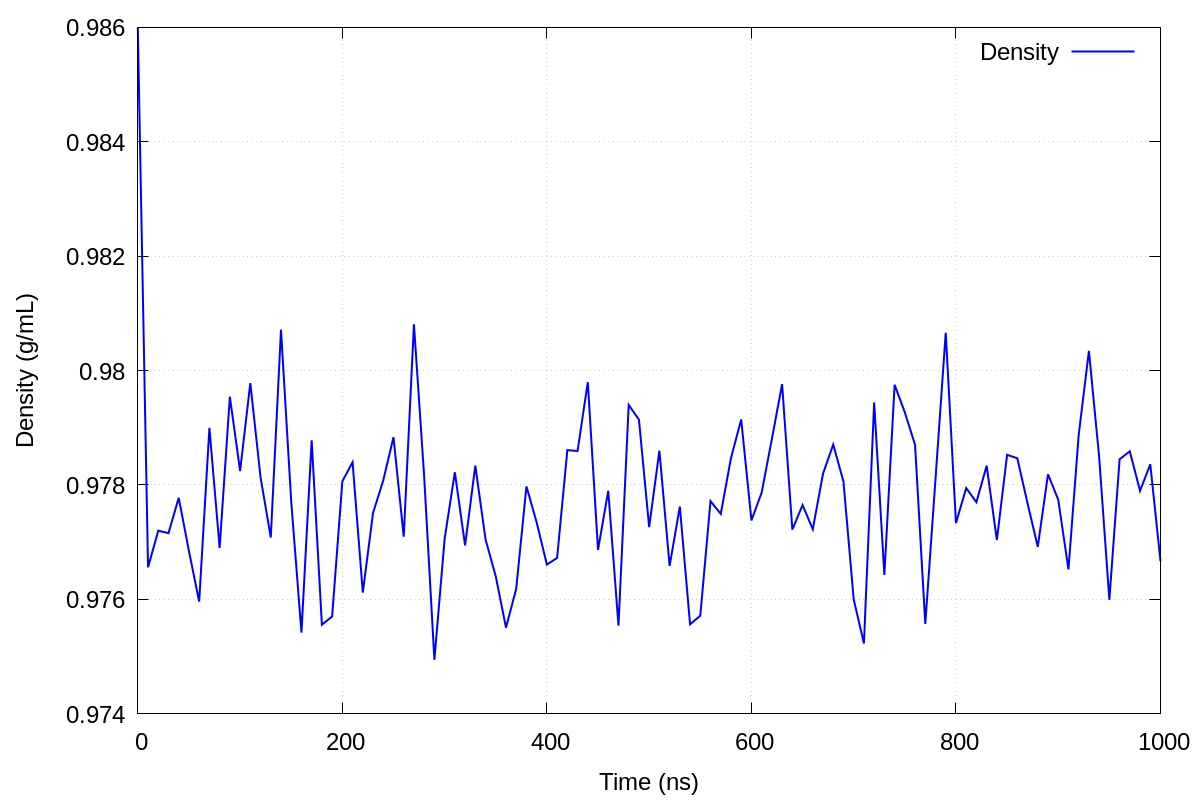


**Figure S5.** Solvent density profile during NPT equilibration of the VCX2 system at 309.65K.

**Table S1.** Root-Mean-Square Deviation (RMSD) and standard deviation (± SD) of the VCX2 protein structure across different time intervals during a 999-nanosecond molecular dynamics simulation.

| Time interval (ns) | Average RMSD (nm) | Standard Deviation (±nm) |
| --- | --- | --- |
| 0-100 | 0.365 | 0.128 |
| 100-200 | 0.537 | 0.079 |
| 200-300 | 0.645 | 0.146 |
| 300-400 | 0.735 | 0.062 |
| 400-500 | 0.777 | 0.076 |
| 500-600 | 0.744 | 0.107 |
| 600-700 | 0.779 | 0.092 |
| 700-800 | 0.783 | 0.068 |
| 800-900 | 0.777 | 0.047 |
| 900-1000 | 0.792 | 0.055 |

**Table S2. Predicted drug-likeness and ADMET profile of PeruNPDB277 (Luteolin-5-O-glucoside).**

| Parameter | Value |
| --- | --- |
| Molecular Weight (MW) | ~448–464 g/mol |
| Topological Polar Surface Area (TPSA) | 190.28 Å² |
| Hydrogen Bond Donors (HBD) | ≥5 |
| Hydrogen Bond Acceptors (HBA) | ≥10 |
| Rotatable Bonds | Moderate |
| Lipinski Rule Violations | ≥2 |
| Gastrointestinal Absorption | Low |
| Bioavailability Score | 0.17 |
| Water Solubility | High |
| P-gp Substrate | No |
| CYP1A2 Inhibition | No |
| CYP2C19 Inhibition | No |
| CYP2C9 Inhibition | No |
| CYP2D6 Inhibition | No |
| CYP3A4 Inhibition | No |
| Overall ADMET Profile | Mixed |

**Table S3.** MM-GBSA energy components for the VCX2–luteolin-5-O-glucoside complex

| Component | Value (kcal/mol) |
| --- | --- |
| Δ_Gbind_ | −35.43 |
| Coulomb | −24.79 |
| vdW | −28.10 |
| Hbond | −3.72 |
| Lipo | −5.62 |
| Solv GB | +26.83 |
| Packing | −0.00 |
